# DFT and QTAIM insights into C_20_ fullerene derivatives as advanced sensors for phencyclidine drug detection in clinical settings

**DOI:** 10.1038/s41598-025-29996-y

**Published:** 2025-12-08

**Authors:** Abdulwahab Alamri, Ahmed Alafnan

**Affiliations:** Department of pharmacology and toxicology, College of Pharmacy, University of Ha’il, Hail, 55476 Saudi Arabia

**Keywords:** Phencyclidine (PCP), Fullerene C20, Density functional theory (DFT), Sensor design, Drug detection, Chemistry, Materials science

## Abstract

The unauthorized use of phencyclidine (PCP) has serious public health consequences, which prompts the need for new sensing approaches that are fast, sensitive and accessible. This study used Density Functional Theory (DFT) and the Quantum Theory of Atoms in Molecules (QTAIM) to examine pristine fullerene C_20_ and two doped ones (AlC_19_ and ZnC_19_) as new sensors for PCP. Geometry optimization and analyses of the molecular electrostatic potential (MEP), electronic properties (HOMO-LUMO gap; chemical potential, electrophilicity-based charge transfer), and sensing performance (adsorption energy, recovery time and electrical conductivity) were performed. Results illustrate that doping significantly changes the electronic and structural properties of the C_20_ framework. Although pristine C_20_ and ZnC_19_ have limited potential, AlC_19_ is promising as a multifunctional material. AlC_19_ has the strongest interaction with PCP, with an adsorption energy (Eads) of -49.44 kcal.mol^-1^, demonstrating excellent potential to remove PCP in adsorbed form. As an electrochemical sensor, AlC_19_ showed a large increase in electrical conductivity (from 2.71 × 10^9^ in the pristine AlC_19_ to 2.77 × 10^9^ in the AlC_19_@PCP complex) and a long recovery time after PCP binding, making it ideal for disposable sensor application (strong and irreversible binding). Furthermore, AlC₁₉ showed exceptional performance as a colorimetric sensor, exhibiting a significant shift in the UV-Vis absorption maximum (from 470 nm to 524 nm) after complexation with PCP. Both NBO and QTAIM analysis revealed that AlC_19_@PCP exhibits a very strong donor-acceptor interaction and a moderate hydrogen-bond-like character, which contributed to its strong performance. All of the above establishes that AlC_19_ is an effective disposable electrochemical sensor, a colorimetric sensor, and an effective adsorbent of PCP that can be further utilized to develop a multi-functional sensing system that could allow for detection of the drug and remediation of the environment.

**Supplementary Information:**

The online version contains supplementary material available at 10.1038/s41598-025-29996-y.

## Introduction

 Phencyclidine (PCP) is a surgical anesthetic drug that was discontinued from human medical use due to its severe and unpredictable side effects, including hallucinations, agitation, and violent behavior. Despite this, PCP found its way into illicit drug markets and has since been used recreationally for its mind-altering effects. The drug works by disrupting the action of glutamate, a neurotransmitter involved in perception, pain response, and memory, leading to a detachment from reality and a distorted sense of self and surroundings^1,2^. Identifying PCP is critically important for several reasons. First, its use poses significant health risks, including psychological distress, cognitive impairment, and physical harm. Users may experience extreme anxiety, paranoia, and even psychosis, which can lead to dangerous behaviors that endanger both the user and others. Additionally, PCP is often mixed with other substances, such as cannabis or embalming fluid, which can exacerbate its harmful effects and complicate medical treatment in cases of overdose or adverse reactions. Accurate identification of PCP is also essential for law enforcement and public health efforts to monitor and control its distribution, as well as for healthcare providers to diagnose and treat individuals who may be under its influence. Early detection and intervention can help mitigate the drug’s impact on individuals and communities, reducing the associated social and economic costs^3–5^. Phencyclidine (PCP) is commonly identified through various techniques, including immunoassay screening tests (i.e., urine drug tests), gas chromatography-mass spectrometry (GC-MS), liquid chromatography-mass spectrometry (LC-MS), and infrared spectroscopy. Because immunoassays are usually quick and simple to administer, they are often performed first as screening tests compared to more complex confirmatory methods. Conversely, GC-MS and LC-MS are more reliably considered gold standard confirmation tests, and can identify low levels of PCP with high confidence. Infra-red spectroscopy is another option to assess the chemical structure of a drug that might contain PCP^6–8^.

However, these methods have notable disadvantages. Immunoassays, while convenient, can produce false positives or negatives due to cross-reactivity with other substances, limiting their reliability. Confirmatory methods like GC-MS and LC-MS, though highly accurate, require specialized equipment and trained personnel, which may not be readily available in all settings, particularly in resource-limited areas or during emergency situations. Additionally, these techniques are time-consuming and costly, making them less practical for rapid on-site identification. Infrared spectroscopy also demands expertise and specialized equipment, further restricting its accessibility^9,10^. These limitations highlight the challenges in identifying PCP, particularly in environments where immediate and accurate detection is critical but resources or expertise are lacking.

Given the limitations of current PCP detection methods (reliance on specialized equipment, expert operators, and significant resources) the need for an accessible, user-friendly method is increasingly urgent. Electrochemical and colorimetric sensors offer promising field-based solutions. Electrochemical sensors provide high sensitivity and selectivity, while colorimetric sensors enable rapid, visual detection without requiring expertise. Both are portable, cost-effective, and suitable for on-site use^11,12^. Nanostructures, particularly carbon-based materials like carbon nanotubes, graphene, and fullerenes, are widely used in sensor design due to their unique properties. Among fullerenes, C_20_ is notable for its small size, high reactivity, and strained structure, which enhance its interaction with target molecules like PCP. Its superior electronic properties and increased surface area improve sensitivity and detection efficiency, making it a promising candidate for advanced PCP sensors^13,14^. Also, the application of C20 fullerene in the field of drug detection and delivery has been highlighted in recent papers. For example, M. R. Jalali Sarvestani et al. showed that C20 has significant potential in the detection of amitriptyline^15^. Similarly, N. Khalaj Zeighami et al. highlighted the dual role of C20 as a sensor and adsorbent for the drug aflatoxin^16^. Also, A. Alhowyan et al. showed that doped C_20_ nanospheres exhibit significant sensitivity to the presence of the drug ecstasy in the environment^17^. Each of these literatures not only highlight the role of C_20_ as a promising material for advanced sensing and drug delivery systems, but also emphasize the effect of doping in improving the electronic and quantum properties of C_20_.

These enhancements make doped C_20_ a promising candidate for advanced sensors, particularly in fields requiring high sensitivity and selectivity, such as environmental monitoring, biomedical diagnostics, and drug delivery systems^18^. Given the critical need for reliable and accessible methods for phencyclidine (PCP) detection and the demonstrated potential of fullerene C_20_ as a highly sensitive material, this study investigates the capability of C_20_ and its doped forms with zinc (Zn) and aluminium (Al) as sensors for PCP detection. Doping C_20_ with zinc and aluminium has been proposed in various studies as an excellent solution to improve the electronic properties of C_20_, as emphasised in one of the most recent works by Huwaimel et al.^19,20^. Given the limitations of laboratory methods, we used computational chemistry/physics methods such as density functional theory (DFT) and quantum theory of atoms in molecules (QTAIM) to investigate the interaction of pristine C_20_ and its doping forms with PCP^21,22^. We hope that the results of this study can pave the way for the synthesis of new adsorbents/sensors for rapid and accurate detection of PCP.

## Computational section

**Fig. 1:**
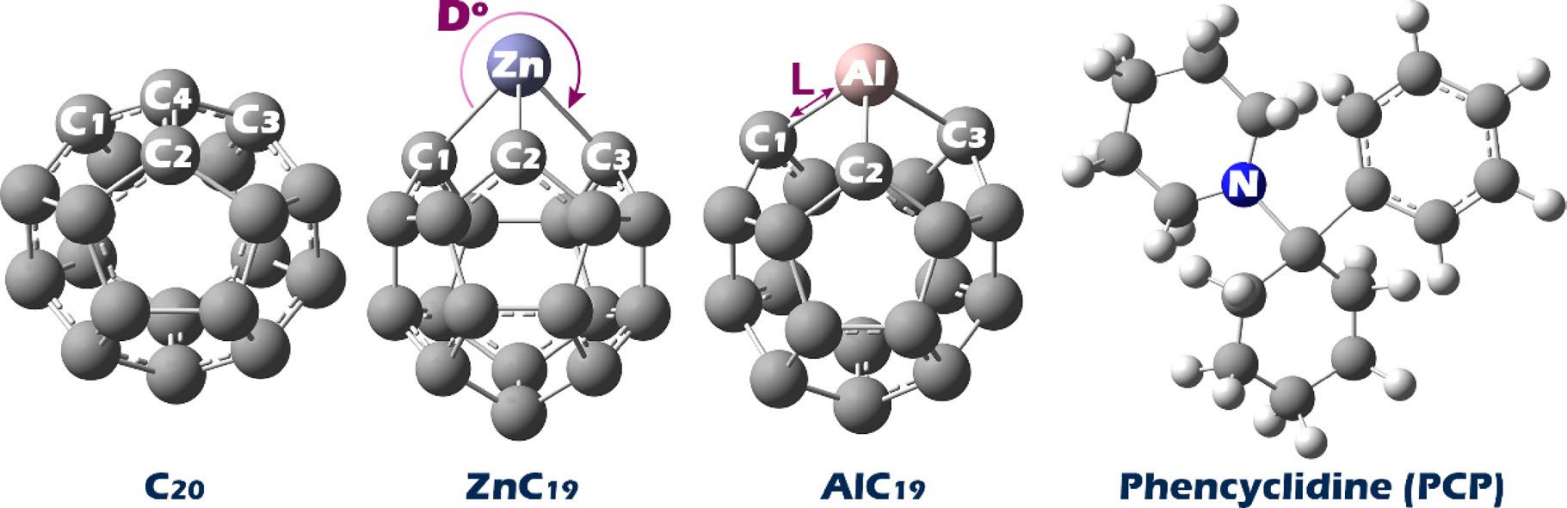
Optimized geometry of PCP, pristine C_20_, and its doping forms with Al and Zn.

First, each of the studied structures (PCP, C_20_, ZnC_19_ and AlC_19_) was designed using GaussView software (see Fig. 1) and then their geometric structure was optimized using Gaussian 09 W software (the full XYZ coordinates of these structures are reported in the Supplementary Data (Table S1)). The geometric structure optimization was performed using the DFT/WB97XD/6–311 + G(d, p) computational code and in the aqueous solvent phase using the continuum polarizable quasiconductor model (CPCM) to simulate physiological conditions^23,24^. The WB97XD functional was chosen because it includes empirical dispersion corrections, which are essential for the accurate description of non-covalent interactions, especially in systems involving π-stacking or van der Waals forces, as expected in fullerene-based structures. No imaginary frequencies were observed in the frequency calculations, indicating the stability of the designed structures. Time-dependent DFT theory (TD-DFT) was also used to investigate the UV spectrum. Then, the UV plot was drawn using GaussSum software. This software was also used to display the density of states (DOS) plot to visually explain the energy gap (HLG).

The stability of the designed systems was evaluated through cohesive energy calculations, as defined in Eq. (1).

1 $$\:{{E}}_{{C}{o}\varvec{h}}=-\left({{E}}_{{t}{o}{t}{a}{l}}-\sum_{{i}}{{n}}_{{i}}{{E}}_{{i}}\right)/{n}$$

Etotal: total energy of the structure; ∑: sum of total energies of atoms ( is the energy of the atom and is the number of atoms of type ) and : total number of atoms in the system^25^.

Reactivity descriptors such as energy gap (HLG), chemical softness (S), chemical hardness (η), chemical potential (µ) and charge transfer parameters such as maximum charge transfer amount (ΔNmax) and electrophilicity-based charge transfer (ECT), were calculated according to Eq. 2 to 7, respectively^26,27^.

2 $$\:\text{HLG}=\left|\text{E}_{\text{HOMO}}-{\text{E}}_{\text{LUMO}}\right|$$

3 $$\:{\upeta\:}=\raisebox{1ex}{$(-{\text{E}}_{\text{H}\text{O}\text{M}\text{O}}-(-{\text{E}}_{\text{L}\text{U}\text{M}\text{O}}\:\left)\right)$}\!\left/\:\!\raisebox{-1ex}{$2$}\right.$$

4 $$\:{\upmu\:}=-(-{\text{E}}_{\text{H}\text{O}\text{M}\text{O}}+(-{\text{E}}_{\text{L}\text{U}\text{M}\text{O}}\left)\right)/2$$

5 $$\:\text{S}=1/2{\upeta\:}$$

6 $$\:{\varDelta\:\text{N}}_{\text{max}}=-{{\mu}}\left/{{\eta}}\right.$$

7 $$\:\text{ECT}={\left({{\Delta\:}\text{N}}_\text{max}\right)}_{{\alpha}}-{\left({{\Delta\:}\text{N}}_\text{max}\right)}_{{\beta}}$$

E_HOMO_ and E_LUMO_ represent the energy of the highest/lowest occupied/unoccupied electron orbitals, respectively.

ΔNmax represents the maximum charge transfer, with α for the complex (C_20_@PCP, ZnC_19_@PCP, and AlC_19_@PCP), and β for the sensor in the absence of PCP (C_20_, AlC_19_, and ZnC_19_). A positive ECT means the sensor donates electrons to furan, while a negative ECT indicates electron transfer from PCP to the sensor^28^.

The sensing mechanism of each designed structure was calculated and evaluated using key parameters including adsorption energy (Eads), recovery time (τ), and electrical conductivity (σ) (Eqs. 8–10).

8 $$\:{\text{E}}_{\text{ads}}={\text{E}}_{\left(\text{R}-\right)\text{C}20@\text{P}\text{C}\text{P}}-\left({\text{E}}_{\text{P}\text{C}\text{P}}+{\text{E}}_{\left(\text{R}-\right)\text{C}20}\right)+{\text{E}}_{\text{B}\text{S}\text{S}\text{E}}$$

9 $$\:{\tau\:}={\text{V}}_{0}^{-1}\times\:\text{exp}\left(-\frac{{\text{E}}_{\text{a}\text{d}\text{s}}}{{\text{k}}_{\text{B}}\text{T}}\right)$$

10 $$\:{\upsigma\:}={\text{A}}{\text{T}}^{3/2}{\text{e}}^{(-\text{H}\text{L}\text{G}/2\text{K}\text{T})}$$

In Eq. 8, E(R-)C_20_@PCP is the total energy of the PCP-sensor complex, EPCP is the energy of the isolated PCP, and E(R-)C_20_ is the sensor energy, including the basis set superposition error (EBSSE) correction, which is calculated by calculating the E_BSSE_ value using “counterpoise = 2” in the output file of the optimized structures. In Eqs. 9 and 10, V0 is the excitation frequency (usually 10^12^ s^− 1^), kB is the Boltzmann constant, T is the temperature (usually taken to be 298 K), and A is the Richardson constant (6 × 10^5^ A.m^− 2^.K^− 2^)^29,30^.

Finally, the QTAIM analysis data and the properties of atoms at the bond critical point (BCP) were analyzed using AIM2000 software^31^.

These calculations provide a detailed analysis of the electronic and structural properties of C20 fullerene and its related derivatives under the presence/absence of PCP.

## Results and discussion

### Bond lengths and bond angles

The bond length and bond angle are critical parameters in molecular design. Deviations from ideal bond lengths and angles can induce strain, alter orbital overlap, and affect conjugation in π-systems, ultimately influencing chemical behavior. When a doping atom (e.g., Zn or Al in C_20_) is introduced, it modifies the local electronic environment, leading to changes in bond lengths and angles due to differences in atomic size, electronegativity, and hybridization. These distortions displace π-electrons, disrupting conjugation and altering charge distribution, which can enhance conductivity, modify optical properties, or induce new reactivity^32^. For this purpose, the changes in bond length and bond angle for pristine C_20_ before/after doping with Al and Zn were reported in Table 1.

**Table 1:** Calculated values ​​of bond lengths (L) and bond angles (D) between some important atoms in the designed structures.

Structure	Bond lengths (Å)		Bond angles (°)	
C_20_	C4-C1	1.43	C1-C4-C2	108.61
	C4-C2	1.43	C1-C4-C3	109.43
	C4-C3	1.43	C2-C4-C3	108.60
AlC_19_	Al-C1	1.92	C1-Al-C2	89.83
	Al-C2	1.92	C1-Al-C3	94.01
	Al-C3	1.92	C2-Al-C3	94.00
ZnC_19_	Zn-C1	2.07	C1-Zn-C2	80.23
	Zn-C2	2.07	C1-Zn-C3	80.26
	Zn-C3	2.07	C2-Zn-C3	80.26

In pristine C20, the C-C bond lengths (1.43 Å) and bond angles (~ 108°−109°) are consistent with a symmetrical sp^2^-hybridized carbon network, typical of fullerene cages. Doping with Al and Zn significantly changes these parameters. In AlC_19_, the Al-C bonds (1.92 Å) are longer than the C-C bonds, and the bond angles around Al (89°−94°) deviate from the ideal tetrahedral angle, indicating structural distortion. The Zn-doped system (ZnC_19_) shows even more pronounced changes, with Zn-C bonds extending further (2.07 Å) and bond angles sharpening to ~ 80°, reflecting greater strain and weaker orbital overlap compared to Al. These structural modifications disrupt the π conjugation in the fullerene framework, especially in ZnC_19_, and concentrate the π electrons near the doped atom.

### Cohesive energy

**Fig. 2:**
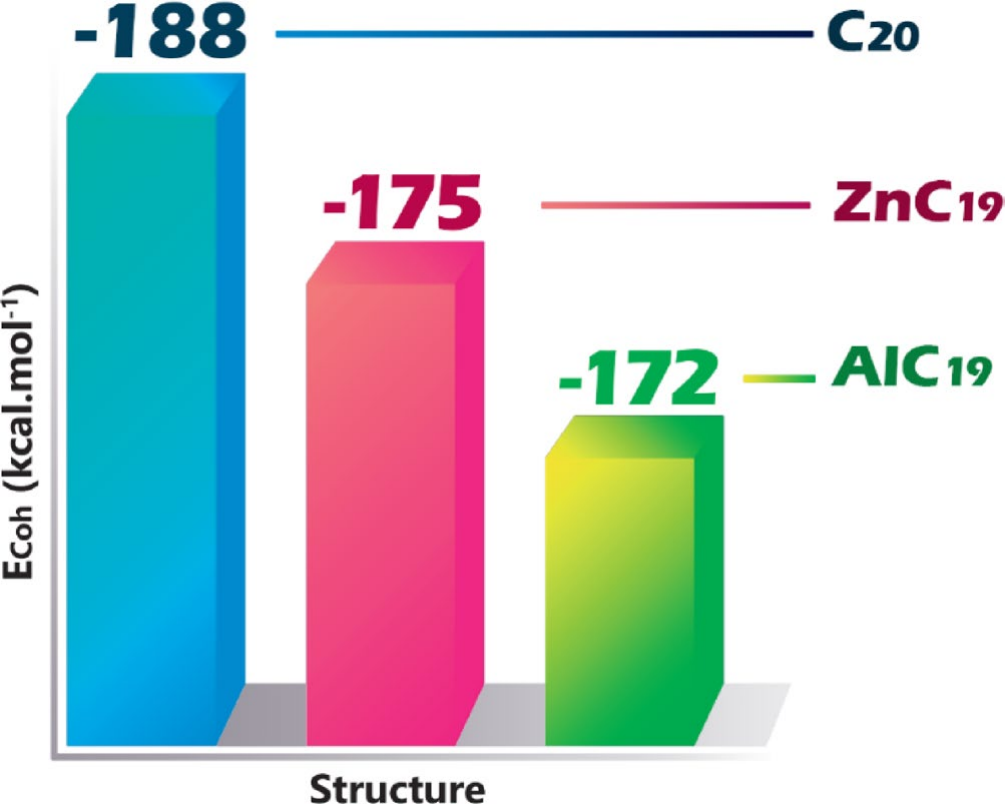
Cohesive energy values for pristine C_20_, AlC_19_ and ZnC_19_.

Cohesive energy is the amount of energy required to break a molecule into its constituent atoms, and higher values ​​indicate stronger interatomic bonds and thus higher stability. Doping can change the bonding interactions and thus the lattice geometry, ultimately affecting the overall stability of the structure^33^. In this regard, the cohesive energy was calculated for each of the structures and reported in Fig. 2.

The bond length/bond angle results are evident in the coherence energy (Table 1). The stability of the pristine C20 cluster is attributed to the perfect tetrahedral bonding geometry suggested by its most negative cohesive energy (−188 kcal/mol). The nearly equal bond lengths of 1.43 Å of the C-C bonds show strong covalent bonds typical of structures that possess bond lengths between C-C double and single bonds, indicating the ideal highly delocalized covalent network. In addition, the bond angles around the central carbon (C4) are the ideal bond angle of sp^3^ hybridization (approximately 109°). Tetrahedral bond lengths and bond angles produce the optimal bond angle and minimal bond strain, leading to an overall maximal and thus highest cohesive energy.

In contrast, the significant destabilization observed in the AlC_19_ structure (−172 kcal/mol) is explained by a severe distortion of this ideal geometry. The Al-C bonds are substantially longer at 1.92 Å, indicating a much weaker and more ionic character compared to the C-C bonds. More strikingly, the bond angles around the aluminum atom are severely constricted to between 90° and 94°, a major deviation from the tetrahedral ideal. This compression introduces significant bond strain into the structure, weakening the overall network and directly accounting for the 16 kcal/mol reduction in cohesive energy compared to C_20_. The aluminum atom disrupts the sp³ carbon network, forming bonds that are both longer and more strained.

The ZnC_19_ structure presents an intermediate case in both stability and geometry. Its cohesive energy (−175 kcal/mol) is higher than that of AlC_19_, and this is consistent with its structural parameters. The Zn-C bond length is even longer at 2.07 Å, which typically suggests a weaker interaction. However, the bond angles around the zinc atom are even more constricted, at about 80°. While this indicates significant strain, the electronic configuration of zinc (a full d^10^ shell) may allow for different bonding interactions that are less disruptive to the overall π-system of the carbon cluster. The result is a structure that, while less stable than C_20_, is more stable than AlC_19_. This implies that the nature of the Zn-C interaction, potentially with some multicenter character, compensates for the extreme angular strain more effectively than the Al-C bond, leading to a net better cohesive energy.

The trend in cohesive energy (C_20_ > ZnC_19_ > AlC_19_) can be explained simply as a result of the geometric changes from doping. The pristine carbon cluster has an ideal set of strong bonding. Doping produces bonds that are longer, weaker, and with a considerable degree of angular strain. Aluminum doping produces the most unstable of the three structures. The instability from doping can greatly impact a structure’s reactivity (this effect is addressed in depth in the sections that follow).

To study the process of optimization and analyze the stability of each of the designed structures, the Energy vs. Optimization Step graph and Deviation from Targets vs. Optimization Step graph for each structure were examined, as presented in Figure S1 (in the Supplementary Data). These graphs illustrate the process each system undergoes in the optimization. By examining the energy and deviation from the target value, we were able to follow the convergence properties of the structures. The Energy vs. Optimization Step graph shows how the system’s energy changes in realization of the optimization, indicating an arrival at the stabilized structure. The Deviation from Target vs. Optimization Step graph is informative about how close the structures’ configurations match the target configurations and can be correlated to when a stabilized and optimized configuration was reached. In sum, we had confirmation that the structures shown in Fig. 1 are based on an efficient optimization process resulting in stable and converged geometries.

The energy plot for C_20_ indicates an initial sharp decrease in energy, followed by stabilization near a minimum value. This indicates that the system quickly stabilized after only a few optimization steps, and therefore, the energy is stable. The deviation from targets for C_20_ shows a remarkable resemblance to the energy trends in each run of the optimization process, with a high initial deviation quickly dropping down and stabilizing. This reflects the efficient convergence of the optimization process and that stability is achieved at a relatively early stage in the optimization process.

The energy plot for ZnC_19_ exhibits more fluctuations during the optimization process, with the energy values swinging up and down several times before stabilizing. However, despite these fluctuations, the overall trend is a decrease in energy, which ultimately reaches a stable region after several steps. The deviation from targets plot also shows noticeable fluctuations but eventually stabilizes, though not as quickly as C_20_.

The energy plot for AIC_19_ closely parallels that of C_20_, where energy rapidly decreases then stabilizes at a relatively low value. The deviation from targets also shows a rapid decrease with the structure stabilizing after a few optimization steps. This suggests AIC_19_ has a stability profile similar to C_20_ that converges efficiently with little deviation from the target values.

### Molecular electrostatic potential (MEP) analysis

**Fig. 3:**
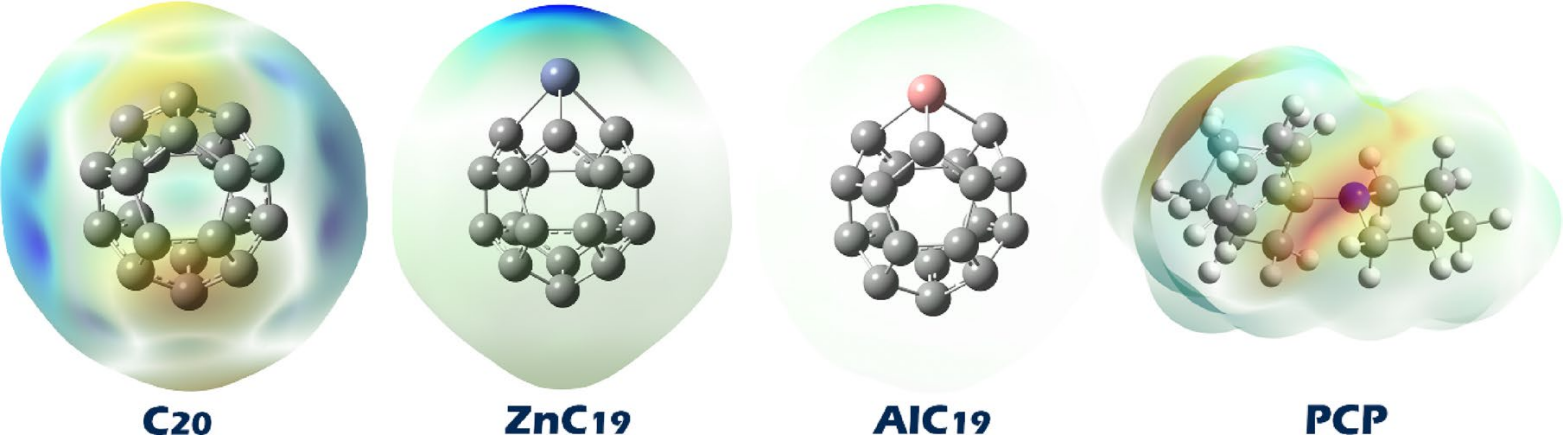
MEP contours for PCP, pristine C20 and its doping forms.

Molecular electrostatic potential (MEP) analysis is a visual and quantitative approach that enables the identification of the most likely sites of intermolecular interactions (Fig. 3)^34^. In MEP maps, red regions indicate areas of high electron density and negative potential that attract electrophiles. Blue regions represent areas of positive potential and electron deficiency that attract nucleophiles. Yellow and green regions show moderate potential associated with weaker interactions^35^.

Given that red regions in MEP maps mark electron-rich (nucleophilic) sites and blue regions mark electron-poor (electrophilic) sites, the red color on the nitrogen atom of PCP identifies its lone pair as the most likely interaction point. In pristine C_20_, the blue color on the cage carbons indicates localized positive potential due to curvature/strain, so complex formation is expected via N(PCP)→C_20_ interactions directed toward those blue carbon atoms (primarily electrostatic with possible n→π* charge transfer). Doping concentrates the electrophilicity: in ZnC_19_, the blue region sits on Zn, and in AlC_19_, it sits on Al, creating clear Lewis-acidic centers. Hence, the most probable adducts are coordination complexes where the PCP nitrogen donates its lone pair directly to the dopant atom (N→Zn in ZnC_19_ and N→Al in AlC_19_).

**Fig. 4:**
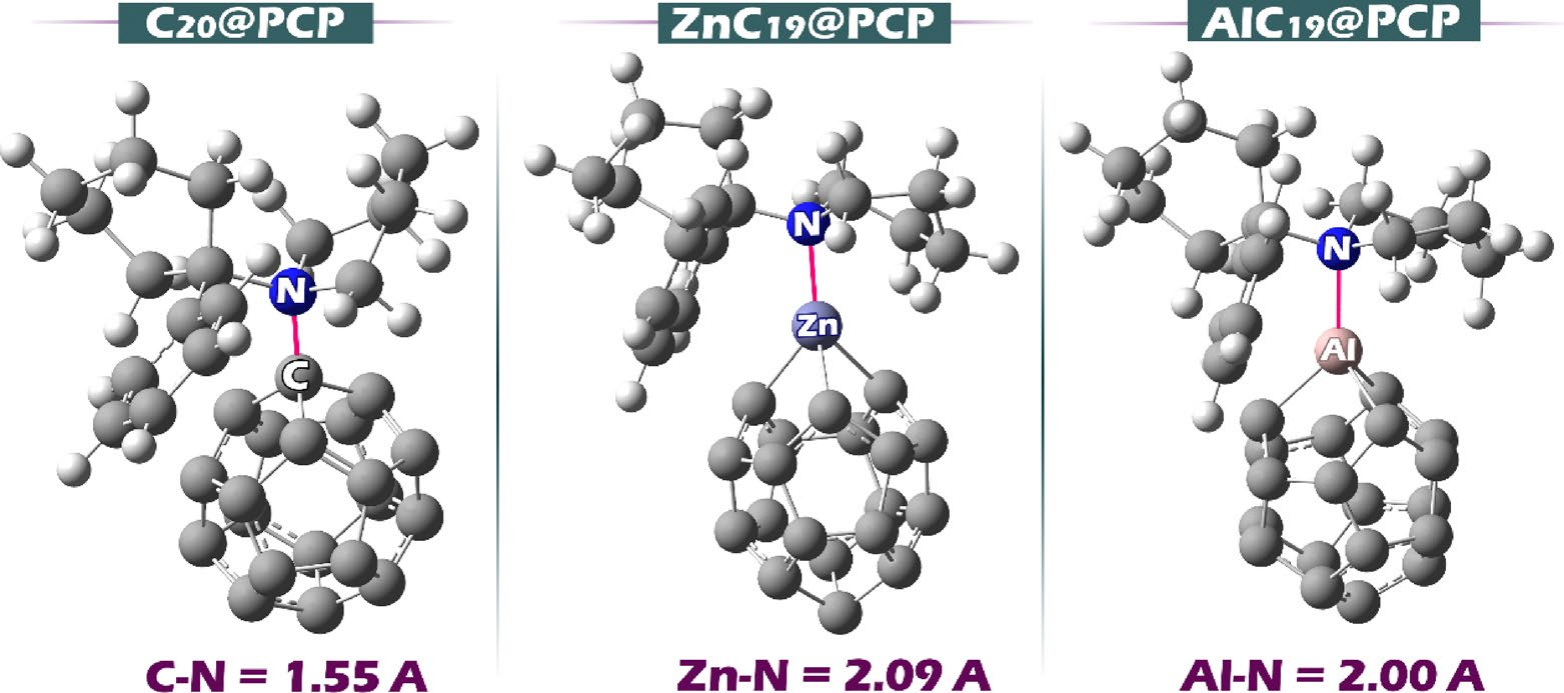
Optimized geometry of each of the complexes designed in this work.

Based on the MEP findings, the geometric structure of each of the complexes was predicted. Figure 4 shows the optimized geometry of each of the designed complexes.

he complex bond lengths follow the order C-N (1.55 Å) < Al-N (2.00 Å) < Zn-N (2.09 Å). The very short C-N distance indicates a relatively strong, partially covalent contact between PCP’s nitrogen and a curved, electrophilic carbon on C_20_. As expected for coordination to metals, the M-N bonds are longer than the C-N bond, but the Al-N bonds, being ~ 0.09 Å shorter than Zn-N, show that Al is the stronger Lewis-acid site toward the nitrogen lone pair, giving tighter binding than Zn.

### Reactivity parameters

In this section, we elucidated the parameters for reactivity and charge transfer. The energy gap (HLG) indicates the difference in HOMO and LUMO energies, which indicates the conductivity and reactivity of a molecule. Chemical softness and hardness indicate how readily the structure will undergo a charge transfer event (softer = more reactive). Chemical potential indicates the likeliness of electrons egressing (donor or acceptor behavior). The maximum charge transfer value (ΔNmax) is just an approximation of how much charge the systems will be able to accept. Electrophilicity based charge transfer (ECT) defines the direction and extent of charge flow between two interacting molecules^36–38^. Each of these parameters were calculated and are reported in Table 2.

**Table 2:** Responsiveness parameter values ​​for each of the designed sensors in the presence/absence of PCP (All values ​​are in eV).

Structure	LUMO	HOMO	HLG	η	µ	S	∆*N*_max_	ECT
C_20_	−1.85	−7.27	5.42	2.71	−4.56	0.18	1.68	**-**
AlC_19_	−1.4	−7.94	6.54	3.27	−4.67	0.15	1.42	-
ZnC_19_	−1.57	−7.03	5.46	2.73	−4.3	0.18	1.57	-
C_20_@PCP	0.05	−5.41	5.46	2.73	−2.68	0.18	0.98	−0.70
ZnC_19_@PCP	−0.93	−7.51	6.58	3.29	−4.22	0.15	1.28	−0.14
AlC_19_@PCP	−0.64	−6.16	5.52	2.76	−3.4	0.18	1.23	−0.34

The data in Table 2 show clear changes in the electronic properties of the sensors after complexation with PCP, indicating significant effects on the reactivity and π-electron mobility. For pristine C_20_, the HLG is 5.42 eV, corresponding to moderate electronic stability and decent π-electron mobility. Upon interaction with PCP, the HLG remains nearly unchanged (5.46 eV), but the chemical potential (µ) shifts from − 4.56 eV to −2.68 eV, suggesting a substantial increase in electron-donating tendency. The marked reduction in ΔNmax (from 1.68 to 0.98) and the negative ECT value (−0.70) confirm that PCP donates charge to C_20_, which corresponds to enhanced π-electron delocalization toward the sensor.

In ZnC_19_, the initial HLG (5.46 eV) increases to 6.58 eV after complexation, with η rising and S decreasing, indicating reduced electronic softness and lower reactivity. The small change in µ (−4.30 to −4.22 eV) and modest drop in ΔNmax (1.57 to 1.28) suggest limited charge redistribution. The negative ECT (−0.14) reveals minimal electron transfer from PCP to ZnC_19_.

**Fig. 5:**
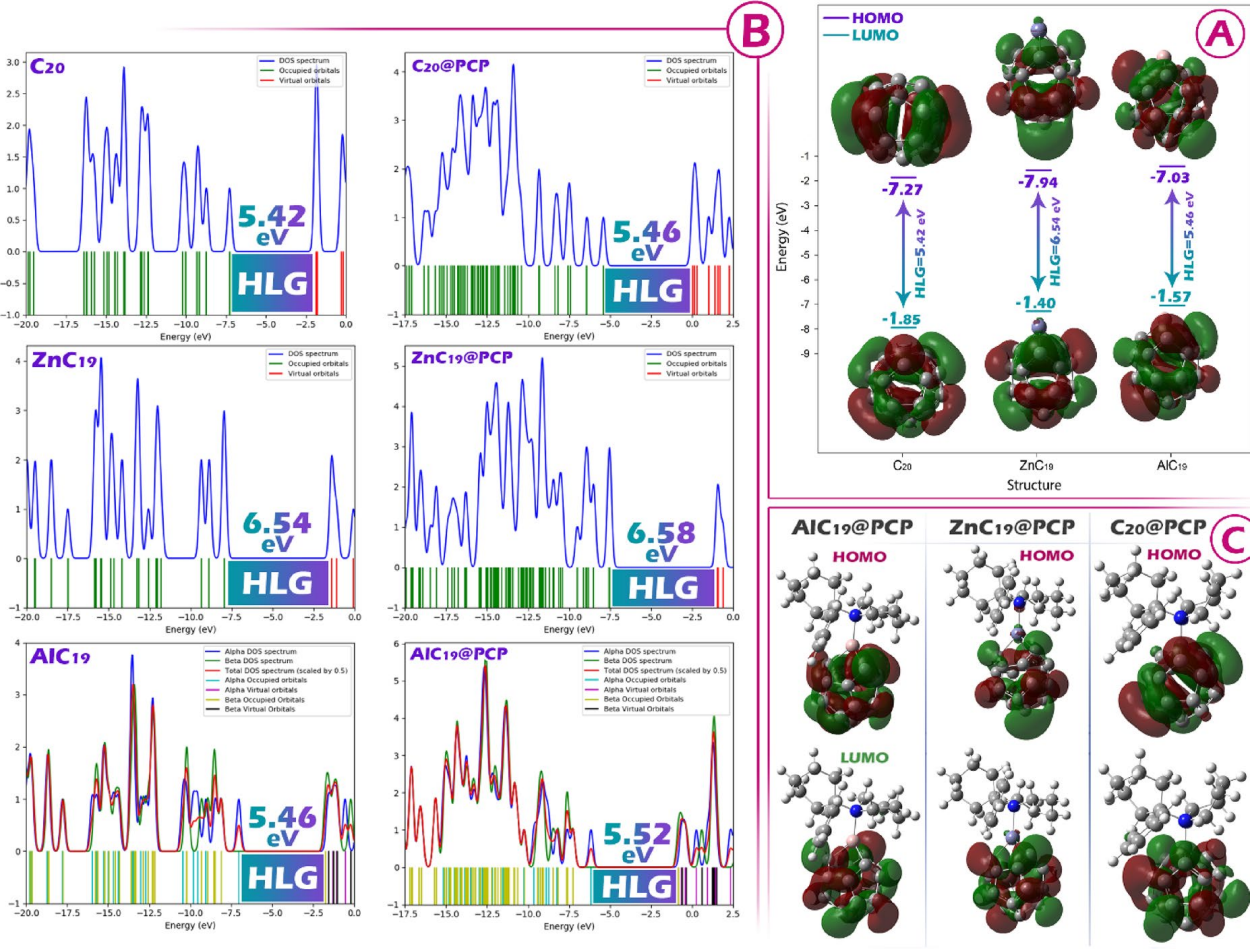
**A**: How the HOMO and LUMO orbitals are positioned and the energy gap in each of the designed sensors. **B**: DOS plot for each of the designed sensors (C_20_, AlC_19_ and ZnC_19_) in the presence/absence of PCP. C: Distribution of HOMO and LUMO orbitals in each of the complexes designed in this work.

For AlC_19_, the HLG decreases from 6.54 to 5.52 eV after binding PCP, with η decreasing and S increasing, which points to higher reactivity and greater π-electron flexibility. The µ value becomes less negative (−4.67 to −3.40 eV), indicating an increased electron-donating capability. ΔNmax decreases moderately (1.42 to 1.23), and the ECT (−0.34) shows notable electron transfer from PCP to AlC_19_, supporting enhanced π-electron mobility compared to ZnC_19_@PCP. The reduction in band gap upon Al-doping (6.54 eV in isolated AlC_19_ down to 5.52 eV in AlC_19_@PCP) is important to discuss in regard to effective doping (Fig. 5A). While the absolute shift may not seem profound, the changes to the electronic structure are significant and, more importantly, exemplify the successful functionalization of the C_20_ building block. The major function of the Al dopant is to provide a Lewis acid site localized to that metal that makes a significant alteration to the local electronic environment, based on the changes to chemical potential (µ), charge transfer (ECT), and ultimately, the optical properties of the material. The reduction seen in HLG is directly related to this pre-engineered reactivity, and is functionally sufficient to facilitate the strong donor-acceptor interaction with PCP. The strong donor-acceptor interaction observed here appeared to induce significant changes to the UV-Vis spectrum, but is a very important criterion in determining an effective colorimetric sensor. Overall, in light of these early results, it is anticipated that aluminum doping is effective, and also an improvement in properties of C_20_ for PCP sensing (of course, this issue needs to be investigated in further detail, and will be presented in the next sections).

The DOS plot is an excellent way to visually represent the distribution of electronic states and the energy gap^39^. In these plots, the separation between the HOMO and LUMO orbitals corresponds to the energy gap, with a wider separation indicating a larger HLG. The DOS plots shown in Fig. 5B clearly show the gaps for each structure, and the measured separations between the HOMO and LUMO levels are consistent with the numerical HLG values ​​reported in Table 2. This agreement confirms the reliability of the calculated orbital energies and supports the observed trends in the electronic properties for the systems studied.

Examining the distribution of HOMO and LUMO orbitals in sensor-analyte complexes is important because it reveals where electrons are most likely to be donated or accepted, directly influencing charge transfer, reactivity, and sensing efficiency^40^. In the designed complexes, both the HOMO and LUMO orbitals are focused on the sensor rather than the analyte (see Fig. 5C). This type of distribution suggests that the electronic structure of the sensor controls the interaction and that changes in conductivity, electron density, or other measurable electronic parameters occur primarily on the sensor. As a result, the sensor maintains its electronic responsiveness, enabling reliable monitoring of analyte binding through measurable changes in properties such as the energy gap or charge distribution.

### Dipole moment

**Fig. 6:**
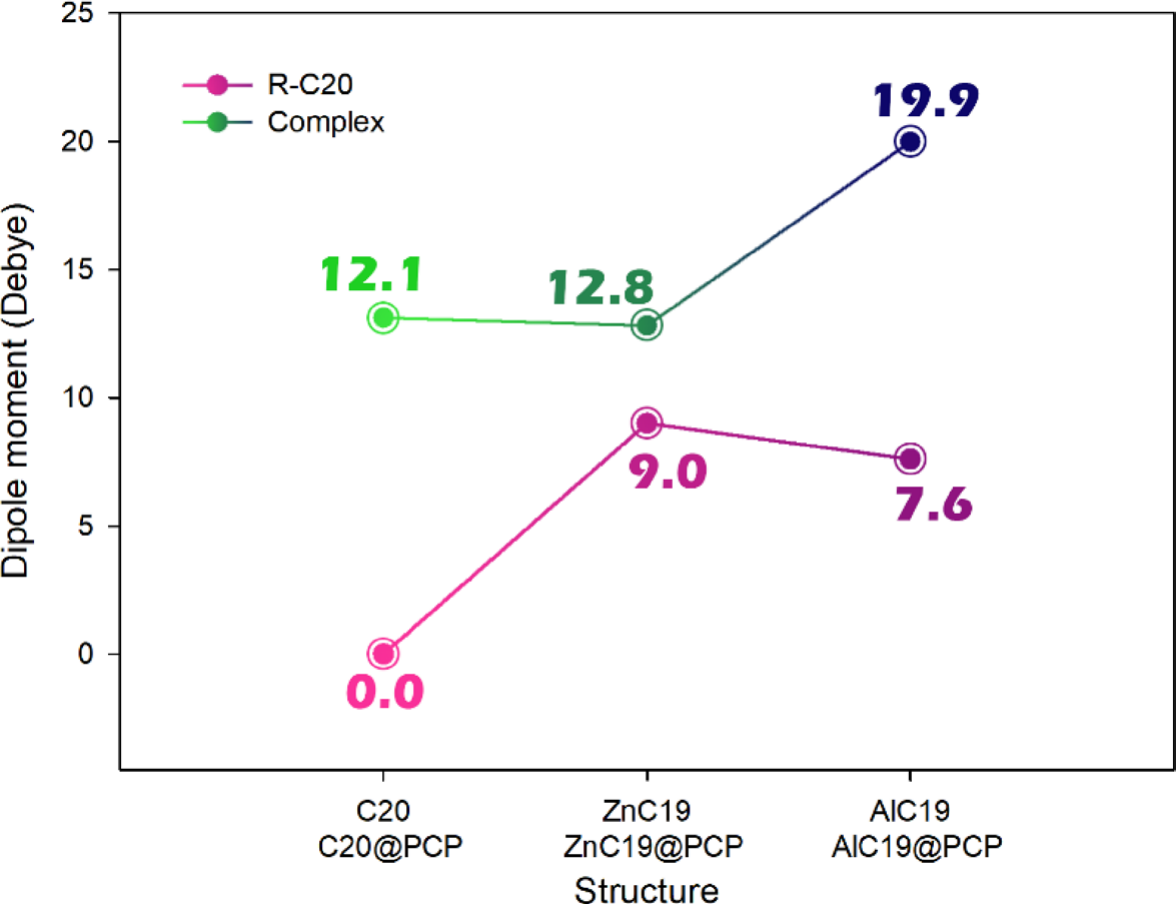
Calculated dipole moment for C_20_ and its doping forms in the presence/absence of PCP.

Considering the dipole moment is important in designing sensor-analyte complexes because it directly influences both their solubility and their sensing performance. A higher dipole moment generally enhances the solubility of the complex in polar solvents, which is crucial for practical applications in solution-based sensing. Additionally, changes in dipole moment upon analyte binding can generate or modify an electrical signal, providing a measurable parameter for detection^41^. Therefore, the dipole moment was calculated for each of the designed sensors in the presence/absence of PCP, and the results were reported in Fig. 6.

The dipole moment results in the Fig. 8 showing significant changes after complex formation with PCP, reflecting strong alterations in charge distribution upon analyte binding. Pristine C_20_ has a dipole moment of 0.0 D, indicating perfect symmetry and no net charge separation. After interaction with PCP, the dipole moment rises sharply to 12.1 D, demonstrating that the analyte breaks the symmetry of the fullerene cage and induces a strong polarization, which is highly beneficial for signal generation in sensing applications.

For ZnC_19_, the initial dipole moment is already high (9.0 D) due to the asymmetry introduced by Zn doping. Upon complexation with PCP, the value increases slightly to 12.8 D, indicating moderate additional polarization. This suggests that ZnC_19_ already possesses an intrinsic polarity, and analyte binding further enhances but does not drastically change the charge separation.

In AlC_19_, the initial dipole moment is 7.6 D, also reflecting inherent asymmetry from doping. However, after PCP binding, the dipole moment increases dramatically to 19.9 D, the highest among all studied complexes. This large increase indicates a strong charge redistribution between AlC_19_ and PCP, suggesting highly effective electronic coupling. Such a large dipole moment change is advantageous because it can produce a stronger electrical signal, improving detection sensitivity.

### Sensor performance

#### Adsorption energy, recovery time, and electrical conductivity

In evaluating the performance of a sensor for analyte detection, absorption energy, recovery time, and electrical conductivity are key parameters. Absorption energy indicates the strength of the interaction between the sensor and the analyte, while recovery time and electrical conductivity determine the speed at which the sensor returns to its initial state and the ability of the sensor to produce a detectable signal, respectively^42,43^. These parameters, which directly affect the sensitivity and accuracy of the sensor, were calculated and reported in Table 3.

**Table 3:** Adsorption energy (Eads), electrical conductivity ($\:\varvec{\sigma\:}$), recovery time ($\:\varvec{\tau\:}$), and basis set superposition error correction (E_BSSE_) values ​​in each of the studied structures.

Structure	E_ads_ (kcal.mol^− 1^)	E_BSSE_ (kcal.mol^− 1^)	$\:\varvec{\tau\:}$ (s)	($\:\varvec{\sigma\:}$) (A.m^− 2^)
C_20_	**-**	**-**	**-**	2.77 × 10^9^
AlC_19_	**-**	**-**	**-**	2.71 × 10^9^
ZnC_19_	**-**	**-**	**-**	2.77 × 10^9^
C_20_@PCP	−19.47	3.74	1.90 × 10^2^	2.77 × 10^9^
AlC_19_@PCP	−49.44	2.64	1.81 × 10^24^	2.77 × 10^9^
ZnC_19_@PCP	−15.58	2.61	2.7 × 10^− 1^	2.71 × 10^9^

Based on the data provided in Table 3, a comparative analysis of the C_20_, AlC_19_, and ZnC_19_ structures in the presence and absence of pentachlorophenol (PCP) reveals critical insights into their performance as both adsorbents and electrochemical sensors.

In the absence of PCP, the pristine structures (C_20_, AlC_19_, ZnC_19_) exhibit no adsorption energy (Eads) or recovery time (τ), as these properties are only defined upon interaction with an adsorbate. Their electrical conductivity (σ) is high and very similar, ranging between 2.71 × 10^9^ and 2.77 × 10^9^ A.m^− 2^, indicating that all three are intrinsically excellent conductors. The introduction of PCP, forming the @PCP complexes, induces significant and divergent changes. The adsorption energy becomes highly negative for all complexes, confirming that the adsorption process is spontaneous and exothermic. However, the strength of adsorption varies dramatically. AlC_19_@PCP exhibits a very strong adsorption energy of −49.44 kcal.mol^− 1^, which is characteristic of a strong chemisorption process. In contrast, ZnC_19_@PCP shows a relatively weak adsorption energy of −15.58 kcal.mol^− 1^, while C_20_@PCP has a moderate value of −19.47 kcal.mol^− 1^.

The ability of AlC_19_ to strongly and persistently adsorb PCP (AlC_19_@PCP) makes it a valuable adsorbent for PCP removal. Such a property can be exploited in environmental applications, and AlC_19_ can be used in filtration or purification systems to adsorb and remove PCP, thereby reducing the release of this toxic compound into ecosystems and reducing its harmful impact on human health and the environment.

The recovery time (τ), which is the time required for the sensor to refresh by releasing the adsorbed PCP molecule, is a crucial parameter for sensor reusability. Here, the results are striking. ZnC_19_@PCP has an exceptionally short recovery time of 0.27 s, suggesting a very weak and highly reversible interaction with PCP. C_20_@PCP has a moderately long recovery time of 190 s. Most notably, AlC_19_@PCP has an astronomically high recovery time of 1.81 × 10^24^ seconds, a value so large that it effectively indicates the adsorption is irreversible for all practical purposes.

**Fig. 7:**
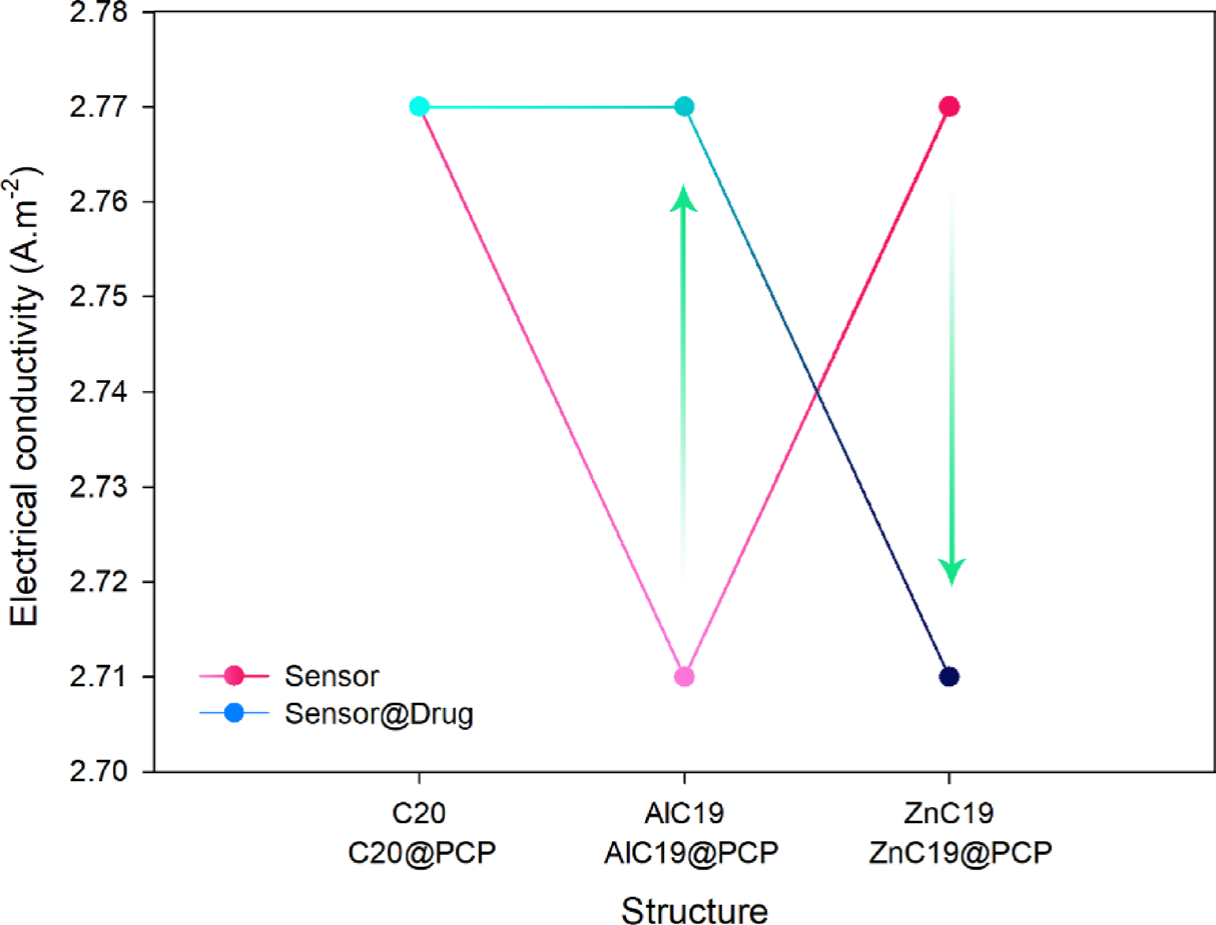
The trend of electrical conductivity changes for each of the designed sensors in the presence and absence of PCP.

According to the results reported, the electrical conductivity (σ) for C_20_ remains practically unchanged after PCP adsorption. In ZnC19, the presence of PCP reduces electrical conductivity, ultimately rendering it impossible to generate an acceptable signal. A decrease in electrical conductivity (For ZnC_19_) upon PCP adsorption indicates the ZnC_19_ becomes more insulating. For effective electrical sensing, the binding event must facilitate charge flow to generate a measurable signal; an inhibitory response provides a weak or counterintuitive signal that is difficult to distinguish from noise, making it an unreliable indicator for detection. However, the AlC_19_ is distinct and shows a more responsive electronic structure. The data indicates that the electrical conductivity of AlC_19_ increases from 2.71 × 10^9^ A.m^− 2^ to 2.77 × 10^9^ A.m^− 2^ upon forming the AlC_19_@PCP complex (See Fig. 7). This change is significant and measurable. This increase in conductivity is a direct consequence of the electronic perturbations induced by the Al dopant. The results of the reactivity descriptors further support these findings (AlC_19_ experiences the largest HLG decrease after PCP attachment (from 6.54 eV to 5.52 eV). According to the relationship for electrical conductivity (Eq. 10), even a decrease in the HLG can lead to an exponential increase in conductivity. The Al dopant facilitates a stronger donor-acceptor interaction with the nitrogen lone pair of PCP, leading to enhanced charge transfer (ECT = −0.34 eV) and a delocalization of electron density within the complex. This improved charge carrier mobility, driven by the narrowed band gap, is the fundamental reason for the observed increase in conductivity. The change in the conductivity of AlC_19_ in the presence of PCP provides a plausible electrical signal that can even be integrated into a more complex sensor structure to expand its range of applications.

#### UV spectrum

**Fig. 8:**
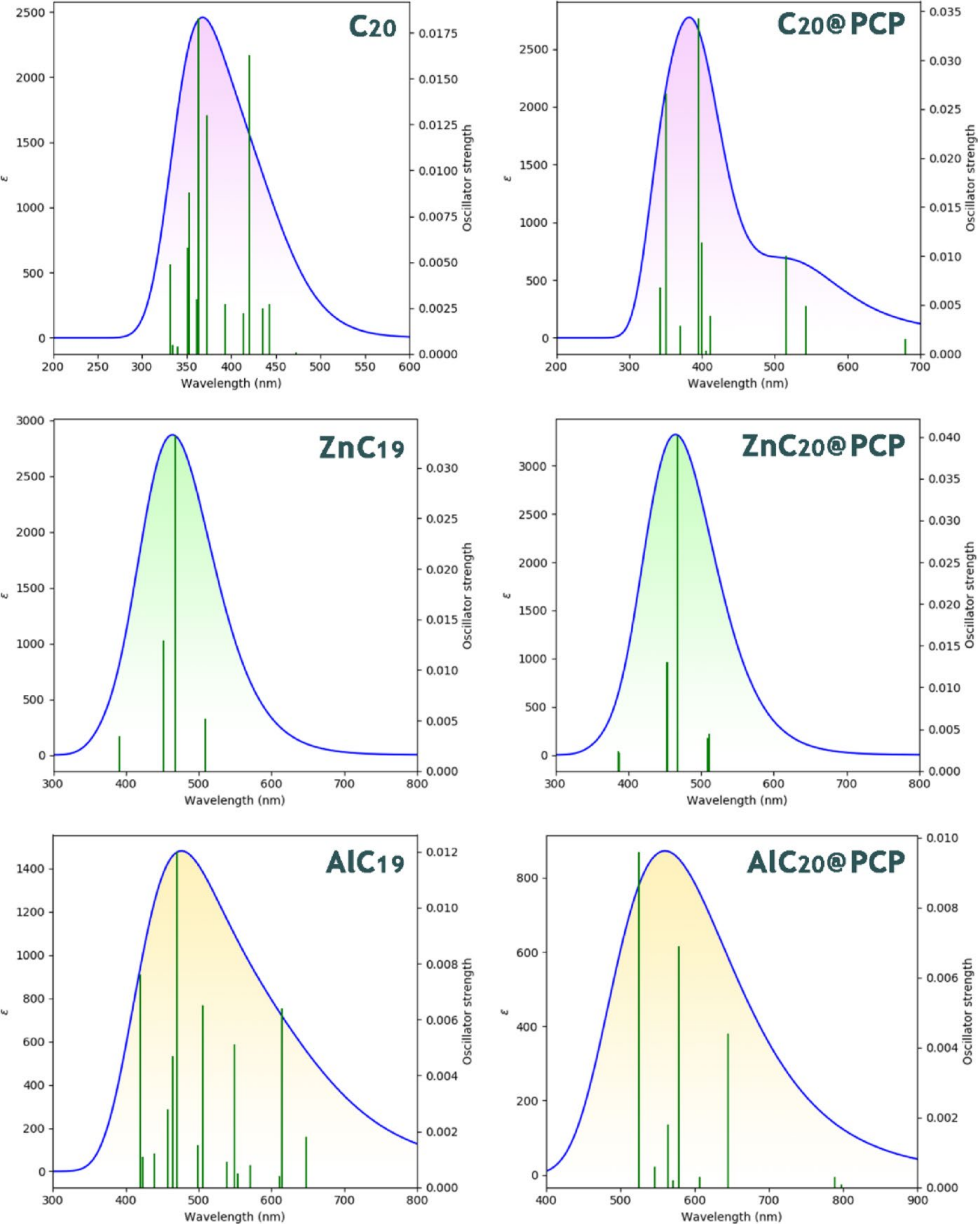
UV spectrum for each of the designed sensors in the presence/absence of PCP.

Since the designed structures cannot function as electrochemical sensors due to the lack of conductivity changes, their potential as colorimetric sensors is evaluated. In this context, the maximum absorption wavelength (λmax), exciton energy (Eex), and oscillator strength (ƒ) are key parameters: λmax indicates spectral shifts upon analyte adsorption, Eex reflects the ease of electronic excitation, and ƒ determines the intensity of absorption^44,45^. Together, these parameters reveal the possibility of using these structures as colorimetric sensors for visual detection of PCP. Each of these parameters was calculated, and the results were reported in Table 4; Fig. 8.

**Table 4:** Values of λmax, Eex and ƒ for pristine C20, ZnC19 and AlC19 and their complexes with PCP.

Structure	λmax (nm)	Eex (Ev)	ƒ
C_20_	363	3.41	0.01
ZnC_19_	467	2.65	0.03
AlC_19_	470	2.63	0.01
C_20_@PCP	394	3.13	0.03
ZnC_19_@PCP	467	2.65	0.04
AlC_19_@PCP	524	2.36	0.009

Comparing the optical data for the three cages before and after PCP adsorption shows clear differences in how the analyte perturbs their electronic structures. C_20_ undergoes a modest bathochromic shift from 363 to 394 nm (Δλ = 31 nm) with a small drop in exciton energy (3.41 → 3.13 eV) and a threefold but still weak increase in oscillator strength (ƒ: 0.01 → 0.03), indicating a detectable but fairly weak optical response. ZnC_19_ shows essentially no spectral shift (467 → 467 nm) and only a tiny increase in ƒ (0.03 → 0.04), so PCP adsorption hardly changes its visible absorption, and it is a poor colorimetric candidate. AlC_19_ exhibits the largest and most significant change on PCP binding: a pronounced red shift from 470 to 524 nm (Δλ = 54 nm), a clear reduction in exciton energy (2.63 → 2.36 eV), and reduction in oscillator strength (ƒ: 0.01 → 0.009).

Although the oscillator strength of AlC_19_@PCP drops slightly from 0.01 before the adsorption of PCP to 0.009 after the adsorption of PCP, this small reduction does not eliminate a detectable color change. Choudhury et al. studied a zwitterionic fluorescent probe that exhibited dramatic solvent-dependent color changes (yellow in toluene to blue-purple in methanol) although the oscillator strength in methanol was low at ƒ = 0.005^46^. As such, a lower ƒ value might lead to a significant observable optical change when the system also exhibits a strong spectral shift in a particular environmental condition (e.g. change in solvent), fitting the context of a large change in color. Therefore, in the case of AlC_19_@PCP, a large bathochromic shift (Δλ = 54 nm) and the reduced exciton energy is producing a decreased ƒ value that is compensated for by the large shifts in both the wavelength of absorption and the visible spectrum, and this indicates that AlC_19_@PCP has a high degree of sensitivity to molecular interactions with PCP.

### NBO and QTAIM analysis

Natural bond orbital (NBO) and QTAIM analysis were performed together in order to better understand the noncovalent interactions governing the sensor-PCP complexes (Table 5)^47^. NBO analysis is crucial for designing sensors as it quantifies the strength and nature of donor-acceptor interactions within the complex. The second-order perturbation energy matrix (E^(2)^) specifically measures the stabilization energy resulting from electron delocalization from a filled donor orbital (e.g., a lone pair (LP) or a π-bond) to an empty acceptor orbital (e.g., a π* or σ* orbital)^48^. A higher value of E^(2)^ indicates stronger orbital interactions, which directly affect the sensing properties^49,50^.

**Fig. 9:**
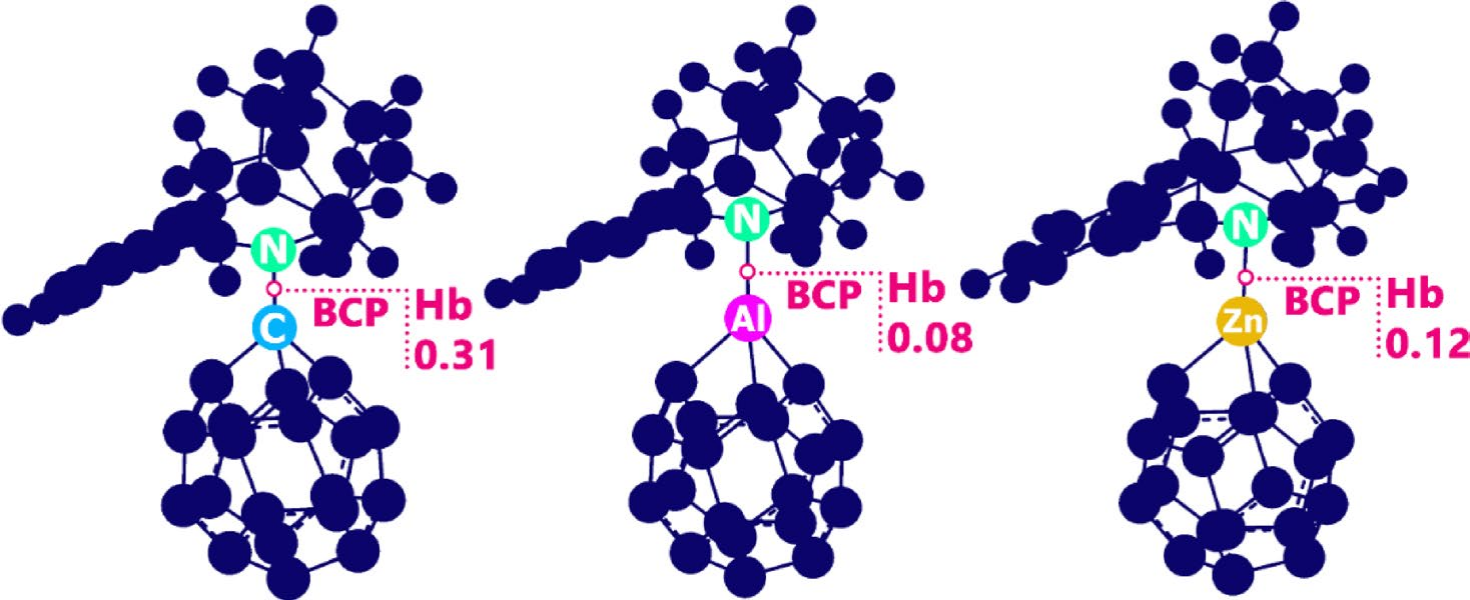
Hb values ​​at BCP.

While NBO reveals the orbital mechanism of interaction, QTAIM characterizes the physical nature of the bond by analyzing the topology of the electron density, ρ(r), at specific points in space^51^. Particular attention is given to Bond Critical Points (BCPs), which exist between interacting atoms and provide key parameters to classify the interaction (Fig. 9). These include: the electron density, ρ(r), which reflects bond strength; the Laplacian, ∇^2^ρ(r), which indicates whether charge is concentrated (negative value, suggesting covalent character) or depleted (positive value, suggesting closed-shell interaction); and the total electron energy density, Hb = V(r) + G(r), which combines potential (stabilizing) and kinetic (destabilizing) energy densities^52^.

Based on these descriptors, the framework established by Rosas and co-workers provides a clear classification for non-covalent interactions: Strong Hydrogen Bond (quasi-covalent): Hb < 0 and ∇^2^ρ(r) < 0; Moderate Hydrogen Bond (electrostatic): Hb > 0 and ∇^2^ρ(r) < 0; Weak Interaction (dispersion-dominated): Hb > 0 and ∇^2^ρ(r) > 0^53^.

A robust and overlapping analysis is developed using NBO, which gauges how electrons translocate between orbitals to yield stabilization, along with QTAIM, which elucidates what type of physical interaction arises from the electron translocation. This analysis enables us to relate the electronic origins of the sensing mechanism to the thermodynamic stability of the complexes and provide a unified interpretation of the sensing performance for C_20_, AlC_19_, and ZnC_19_.

**Table 5:** Calculated values ​​of NBO and QTAIM analysis for the studied complexes.

NBO Analysis							
Complex	Donor (i)	Type	Acceptor (j)	Type	E^(2)^ kcal.mol^− 1^	E(j)-E(i) a.u.	F(i, j) a.u.
C_20_@PCP	C1-C5	$\:\sigma\:$	C4-H33	$\:{\sigma\:}^{*}$	2.12	1.28	0.047
	C45-C48	$\:\pi\:$	C57-C58	$\:{\pi\:}^{*}$	18.94	0.45	0.083
	C44	LP (1)	C45-C48	$\:{\pi\:}^{*}$	58.52	0.21	0.118
ZnC_19_@PCP	C1-C5	$\:\sigma\:$	C11-N30	$\:{\sigma\:}^{*}$	2.51	1.02	0.046
	C23-C25	$\:\pi\:$	C22-C24	$\:{\pi\:}^{*}$	34.17	0.39	0.104
	C53	LP (1)	C5-C59	$\:{\pi\:}^{*}$	1.04	0.19	0.015
AlC_19_@PCP	C1-C5	$\:\sigma\:$	C4-H33	$\:{\sigma\:}^{*}$	2.06	1.29	0.046
	C26-C27	$\:\pi\:$	C22-C24	$\:{\pi\:}^{*}$	38.19	0.39	0.109
	C44	LP (1)	C46-C47	$\:{\pi\:}^{*}$	59.33	0.23	0.130
QTAIM Analysis							
Complex	ρ(r)		∇^2^ρ(r)		V(r)	G(r)	VIR
C_20_@PCP	0.219		0.119		0.094	0.213	0.308
ZnC_19_@PCP	0.076		−0.056		0.086	0.029	0.115
AlC_19_@PCP	0.062		−0.077		0.081	0.003	0.085

For the C_20_@PCP complex, the NBO analysis identifies a significant lone-pair to π* (LP→π*) donation from the fullerene cage to the PCP molecule, evidenced by a substantial second-order stabilization energy (E^(2)^) of 58.52 kcal.mol^− 1^. This suggests a strong orbital-based interaction. However, the QTAIM topology analysis presents a more nuanced reality. The high electron density at the bond critical point (BCP), ρ(r) = 0.219 a.u., combined with a positive Laplacian (∇^2^ρ(r) > 0) and a positive total energy density (Hb > 0), definitively classifies the interaction as weak and physically dominated by dispersion forces. This apparent contradiction is resolved by understanding that the NBO energy reflects a localized orbital interaction, but the overall stability of the complex is governed by the closed-shell, van der Waals-type interaction captured by QTAIM. This overlap of data explains C_20_’s strong adsorption energy but its poor performance as a reversible sensor.

In stark contrast, the AlC_19_@PCP complex demonstrates a perfect synergy between NBO and QTAIM descriptors, outlining the ideal profile for a sensing interaction. The NBO analysis reveals the strongest donor-acceptor interaction in the study, with an E^(2)^ of 59.33 kcal.mol^− 1^ for a key LP→π* transition, supported by the highest orbital overlap F(i, j) value of 0.130 a.u. This indicates exceptional charge delocalization. The QTAIM data directly corroborates this enhanced interaction, but with a different physical origin. The negative Laplacian (∇^2^ρ(r) = −0.077 a.u.) signifies a concentration of electron density at the BCP, a hallmark of interactions with partial covalent or strong electrostatic character. The small positive Hb value classifies it specifically as a moderate hydrogen-bond-like interaction, which is electrostatic in nature. The overlap here is clear: the powerful orbital interaction (NBO) is facilitated by and manifests as a favorable electrostatic stabilization (QTAIM). This combination is the fundamental reason for the change in electrical conductivity in the presence of PCP, the significant charge transfer (ECT = −0.34 eV), and the dramatic change in dipole moment, making it an excellent electrochemical sensor.

The ZnC_19_@PCP complex presents a less effective hybrid profile. Its primary NBO interaction is a π→π* transition with a moderate E^(2)^ of 34.17 kcal.mol^− 1^, which is significantly weaker than the pivotal LP→π* interactions in the other two complexes. While the QTAIM parameters (negative Laplacian, positive Hb) mirror those of AlC_19_ and also classify it as a moderate hydrogen bond, the weaker orbital interactions revealed by NBO create a less responsive system. This overlap of a moderately favorable electrostatic environment (QTAIM) with inferior charge delocalization (NBO) explains why ZnC_19_, despite its structural stability, shows a minimal electronic response and is a poor candidate for sensing.

### NCI analysis

Non-covalent interaction (NCI) analysis illustrates and rationalizes weak interactions, such as hydrogen bonds, van der Waals forces, and steric repulsions. By analyzing the electron density and the gradients of the electron density, NCI reveals the role of these weak interactions on strength of binding, molecular recognition and sensor performance. NCI contour plots make use of reduction density gradient (RDG) to locate regions of interaction, electron density (ρ) to assess strength of interactions, and the sign of the second eigenvalue of the Hessian matrix (sign(λ_2_)ρ) to distinguish attractive, repulsive, and weak dispersion (See Figure S2 in the Supplementary Data)^54–57^.

In the clean C_20_@PCP, we see a separate peak in the negative and almost zero regions of sign(λ_2_)ρ that correspond to attractive interactions like hydrogen bonding or π–π stacking (negative region) and dispersive van der Waals interactions (almost zero region) respectively. In the positive region, although less distinctive, indicates steric repulsions in the structure.

In the case of ZnC_19_@PCP, the overall pattern is similar, but the negative sign (λ_2_)ρ region shows a lower intensity than C_20_. This suggests that these stabilizing non-covalent interactions are weaker in Zn-PCP complexes, and dispersive forces and steric contributions are more relevant. This also corresponds to the less significant charge delocalization identified in NBO analysis and suggests that Zn doping doesn’t really enhance non-covalent stabilization.

AlC_19_@PCP shows more significant attractive interactions, as indicated by the marked features in the negative sign(λ_2_)ρ region. The broad density near zero corresponds to increased van der Waals contributions, while the steric repulsions are minimal. In total, doping with aluminum increases favorable noncovalent interactions that improve the stability and responsiveness of the entire system.

**Fig. 10:**
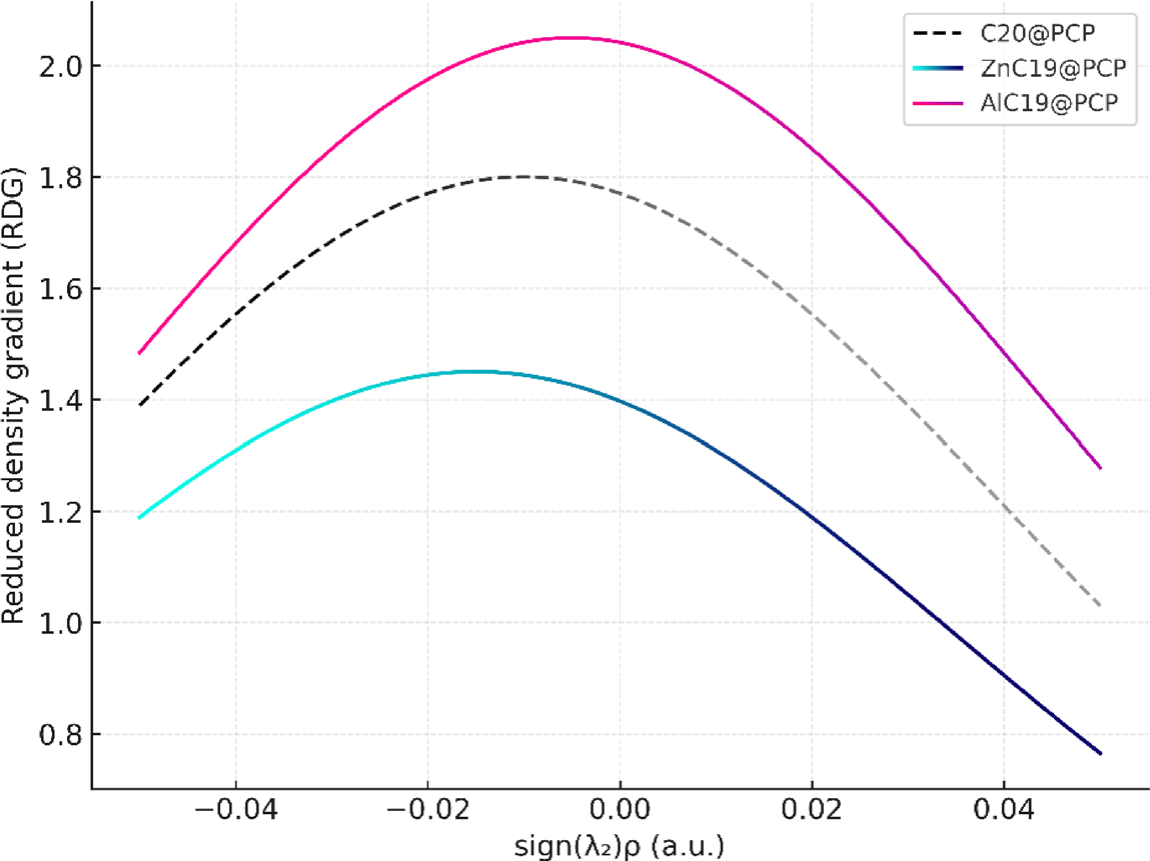
Schematic overlay of NCI profiles for C_20_@PCP, ZnC_19_@PCP, and AlC_19_@PCP complexes.

Figure 10 compares the reduced density gradient (RDG) versus sign(λ_2_)ρ, where negative values indicate attractive interactions, near-zero values correspond to dispersive forces, and positive values represent steric repulsions. The AlC_19_@PCP complex (red) exhibits stronger and broader features in the negative region, confirming enhanced attractive non-covalent interactions, while ZnC_19_@PCP (blue) shows weakened interactions. The pristine C_20_@PCP (black dashed) displays intermediate behavior. These results highlight the reinforcing effect of Al doping and the diminishing influence of Zn substitution on non-covalent stabilization.

## Conclusion

In this work, the interaction of PCP with C_20_, AlC_19_ and ZnC_19_ was studied using DFT, QTAIM theories. MEP, NBO and NCI analyses showed that the doping significantly changes the structural and electronic properties of C_20_ nanocavities in the presence/absence of PCP. Doping also caused significant structural perturbations. The cohesive energy values dropped from − 188 kcal/mol for the pristine but very stable C_20_, to −175 kcal/mol for ZnC_19_, and down to −172 kcal/mol for AlC_19_, indicating more structural strain which result in increased reactivity.

The electronic response to PCP binding is especially pronounced for AlC_19_. There was a large decrease in the HOMO-LUMO gap (HLG) from 6.54 eV to 5.52 eV (1.02 eV), a large negative ECT of −0.34 eV, and an increase in dipole moment from 7.6 D to 19.9 D. In comparison, ZnC_19_ displayed an increase in HLG (5.46 eV to 6.58 eV), a small ECT of −0.14 eV, and a modest increase in diploe moment (9.0 D to 12.8 D). Pristine C_20_ gave an intermediate response with an almost unchanged HLG (5.42 eV to 5.46 eV) but a reasonable ECT of −0.70 eV and increased dipole moment from 0.0 D to 12.1 D related to symmetry breaking.

Sensor-related performance metrics further strengthened the results. Values of adsorption energy (Eads) reflected the interaction type: AlC_19_@PCP exhibited strong chemisorption (−49.44 kcal/mol), while C_20_@PCP showed moderately physiosorbed (−19.47 kcal/mol) interaction, and ZnC19@PCP suggested weak interaction (−15.58 kcal/mol). Thus, recovery time (τ) was extraordinarily long for AlC19@PCP (1.81 × 10^24^ s suggesting irreversibility), reasonable for C_20_@PCP (190 s), and impractically short for ZnC_19_@PCP (0.27 s). For electrical sensing, only AlC_19_ presented a favorable increase in electrical conductivity (σ) upon PCP binding, while ZnC_19_ exhibited a decrease in σ and for C20, the change in σ was negligible.

Similar to the result of colorimetric sensors, the UV-Vis spectral shifts (Δλmax) presented a related narrative. AlC_19_ exhibited a dramatic bathochromic shift at a value of 54 nm (from 470 nm to 524 nm). This value was substantially greater than the larger value of 31 nm, which was observed in C_20_ (363 nm to 394 nm), and without a shift of any value, 0 nm, for ZnC_19_, which remained at 467 nm.

NBO, NCI, and QTAIM analyses strongly support these findings. The NBO results indicate that AlC_19_@PCP exhibited the strongest donor–acceptor interactions, with stabilization energies larger than that of naive C_20_ and far stronger than that of ZnC_19_. The NCI contour plots and RDG analysis verify that AlC_19_@PCP exhibited enhanced attractive non-covalent interactions relative to the weak interactions of ZnC_19_@PCP. In addition, the QTAIM parameters further confirmed these differences, where C20@PCP interactions appeared to arise solely from weak dispersion forces (ρ(r) = 0.219, ∇^2^ρ > 0), but AlC_19_@PCP and ZnC_19_@PCP both exhibit moderate hydrogen-bonding characterized by the customary features of negative Laplacians and small positive Hb values signifying stabilizations, driven by electrostatics. The NBO analysis exhibited that AlC_19_@PCP possessed the strongest donor-acceptor interactions (E^(2)^ = 59.33 kcal/mol), and QTAIM parameters indicates that AlC19@PCP forms a stabilizing electrostatic driven moderate hydrogen bond (∇^2^ρ(r) < 0, Hb > 0).

Finally, this comparative study illustrates that all constructs served a different purpose: ZnC_19_ is ineffective for detecting PCP due to its weak transient binding and lack of an electronic/optical response; pristine C_20_ was tested and shows to be a good reversible physisorption-based sensor with an identifiable optical signal; but AlC_19_ was the most advantageous and multifunctional material. AlC_19_’s strong, specific chemisorption will allow it to serve as an excellent adsorbent for removing PCP, while the structural perturbations (HLG, σ) in the electronic structure and high variation in optical properties (λmax) demonstrate it as a promising candidate for both an electrochemical and colorimetric sensor. This study demonstrates that Al-doped C_20_ is the most promising nanostructure in order to advance rapid and reliable technologies for detection of PCP.

## Supplementary Information

Below is the link to the electronic supplementary material.

Supplementary Material 1

## Data Availability

All data generated or analyzed during this study are included in this published article.
