# Supplementary material for: DFT and QTAIM insights into C20 fullerene derivatives as advanced sensors for phencyclidine drug detection in clinical settings
Source: Sci Rep. 2025 Dec 8;16:481. doi: 10.1038/s41598-025-29996-y (PMC12775048; doi:10.1038/s41598-025-29996-y)
Supplement: Supplementary file 1 — Supplementary Material 1 [file 41598_2025_29996_MOESM1_ESM.docx]

Figure S1 shows the optimization data for the three designed structures (C20, ZnC19, and AIC19). In each graph, the energy and deviation from targets are tracked across optimization steps. For C20, the energy sharply decreases initially and then levels off, while the deviation shows fluctuations. ZnC19 also experiences a sudden drop in energy, followed by irregular increases and decreases in both energy and deviation. AIC19 follows a similar trend, with a sharp drop in energy at the start and a steady decline in deviation, indicating rapid convergence in the optimization process. The overall pattern suggests that the optimization for each compound quickly stabilizes after initial fluctuations.

| 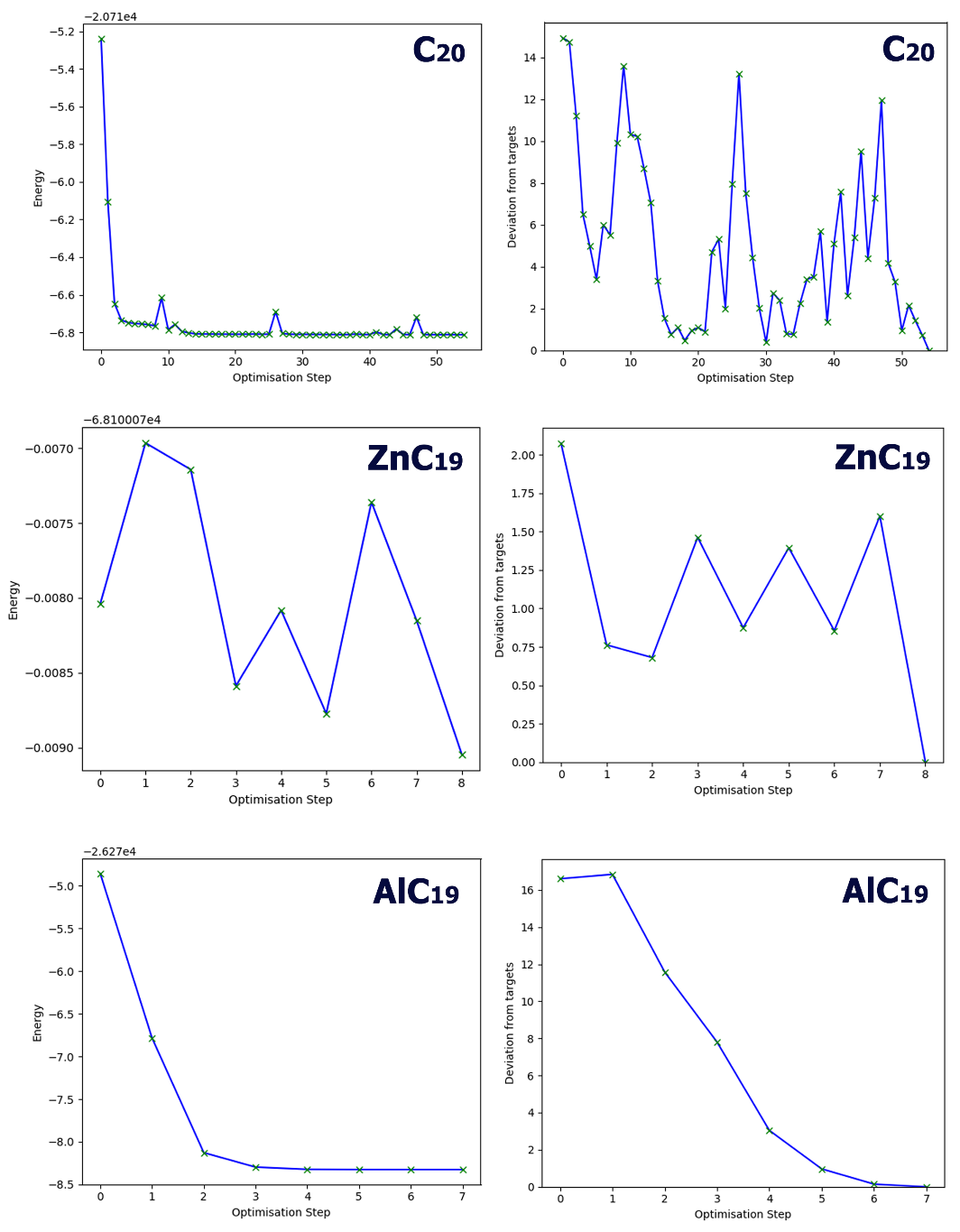 |
| --- |
| Fig S1. Energy vs. Optimization Step (right column) and Deviation from Targets vs. Optimization Step (left column) graphs to examine the optimization steps to achieve the most stable configuration for each of the designed structures. |

The Fig S2 displays reduced density gradients against the sign of λ2ρ for three different compounds: C20, ZnC19, and AIC19. For each compound, the data shows a characteristic "V" shaped structure, with steep peaks near zero and a rapid drop in density gradients as the value of sign(λ2ρ) moves away from zero. The sharp central peak at the origin and the overall spread of points indicate a concentration of points around the central value, suggesting a high degree of symmetry and structure in the data.

| 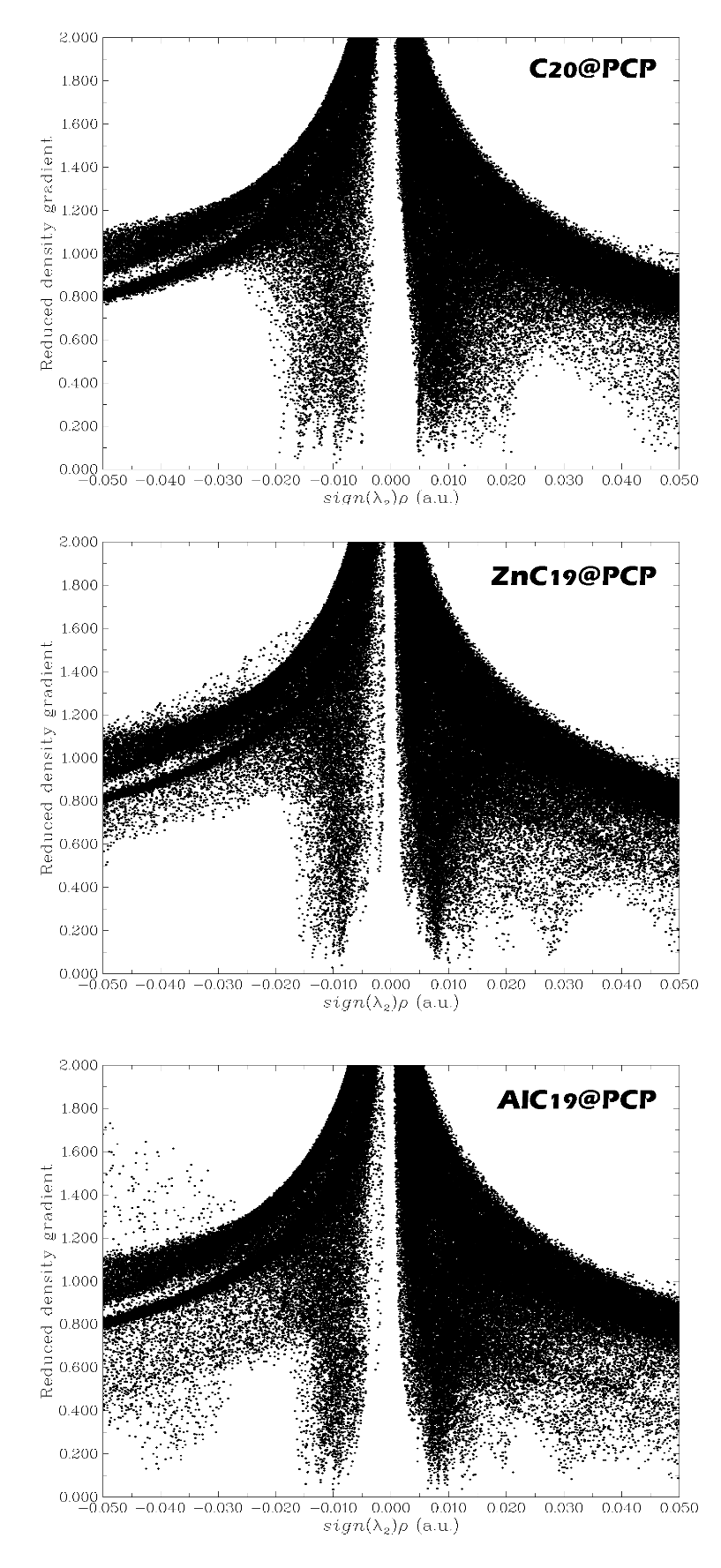 |
| --- |
| Fig S2. The NCI scatter plots of the studied complexes (C_20_@PCP, ZnC_19_@PCP, and AlC_19_@PCP). |

The complete XYZ coordinates of each of the structures designed in this work in the presence and absence of PCP were reported in Table S1.

| Table S1. The full XYZ coordinates of all structures studied in this work. |
| --- |
| ZnC19 |
| %nprocshared=4  # opt wb97xd/6-311+g(d,p) scrf=(cpcm,solvent=water) geom=connectivity out=wfn  Title Card Required  0 1  C -1.23008300 -0.52410100 -1.78903100  C -1.23001600 -1.62666700 -0.91121500  C 0.15304200 -0.05088400 -1.85815600  C 1.06091000 -0.96293600 -1.20891800  C 0.15301100 -1.79964300 -0.46533200  C 0.15201800 1.30358300 -1.32563300  C 1.05905600 1.53016600 -0.22888700  C -1.23127200 1.60172100 -0.95301900  C -1.23170600 1.81085200 0.44068700  C 0.15124300 1.63512100 0.88524700  C -2.07338400 0.47601700 -1.21225800  C -2.72809100 -0.00070500 -0.00059200  C -2.07421500 0.81085200 1.01822800  C -2.07312500 -1.28832100 0.19283900  C -1.23068100 -1.28752700 1.34799100  C -1.23131100 0.02398100 1.86360600  C 0.15174900 0.49670000 1.79159300  C 1.06023600 -0.56545400 1.43914300  C 0.15254600 -1.58382700 0.97363200  Zn 2.44801400 0.00021500 0.00001500  1 2 1.5 3 1.0 11 1.5  2 5 1.0 14 1.5  3 4 1.5 6 1.0  4 5 1.5 20 1.0  5 19 1.0  6 7 1.5 8 1.0  7 10 1.5 20 1.0  8 9 1.5 11 1.5  9 10 1.0 13 1.5  10 17 1.0  11 12 1.0  12 13 1.0 14 1.0  13 16 1.5  14 15 1.5  15 16 1.5 19 1.0  16 17 1.0  17 18 1.5  18 19 1.5 20 1.0  19  20 |
| ZnC19@PCP |
| %chk=PCP-ZnC19.chk  %mem=40GB  %nprocshared=40  # opt wb97xd/6-311+g(d,p) scrf=(cpcm,solvent=water) geom=connectivity  Title Card Required  0 1  C 2.03333300 2.62142800 0.35608400  C 2.01733800 1.30663100 -1.67422600  C 1.59259900 2.64684200 -2.29161800  C 0.46413900 3.31585100 -1.51886300  C 0.91328700 3.57446300 -0.07208500  H 2.09090600 2.58469800 1.44256100  H 3.06789500 1.14163600 -1.91470500  H 2.45219500 3.32341800 -2.32394700  H 0.17337800 4.25086900 -2.00206800  H 0.06408700 3.48627800 0.60894200  C 2.81449300 0.22802400 0.46213300  C 4.24057200 0.81421500 0.55607800  C 2.34441900 -0.04523100 1.92014100  C 5.26120900 -0.18243400 1.14200700  H 4.59486800 1.19780200 -0.40325200  C 3.48774600 -0.37492900 2.89730900  H 1.81264200 0.82993300 2.29890800  C 4.62933100 -1.10317100 2.19638900  H 5.72257500 -0.78059900 0.35397000  H 3.08856300 -0.97215700 3.72051400  H 4.23986600 -2.00964600 1.72110600  C 2.79662500 -1.05344800 -0.40154700  C 3.76116300 -1.27361700 -1.39049100  C 1.79812100 -2.01926000 -0.26300900  C 3.73023400 -2.40100800 -2.20104900  C 1.75247900 -3.14510200 -1.07802900  C 2.72185200 -3.34567600 -2.05120800  H 0.95385900 -3.86611600 -0.94291900  H 2.69379700 -4.22526500 -2.68429800  N 1.83189900 1.23926800 -0.17792100  H 1.44781000 0.48652900 -2.11097200  H 1.30357400 2.46144200 -3.32835600  H -0.42241800 2.67485700 -1.53652200  H 1.30391200 4.59043100 0.03029200  H 2.99293100 2.99773800 -0.00763600  H 4.55559100 -0.55725200 -1.55265900  H 4.49753900 -2.53619200 -2.95503200  H 1.02131800 -1.90763700 0.48016900  H 4.19709800 1.67673200 1.22269000  H 6.06476300 0.40240400 1.59901200  H 5.38473200 -1.42426900 2.91784800  H 3.87574200 0.54764000 3.34319600  H 1.61828200 -0.85471800 1.93356100  C -3.15711100 -2.15347600 0.85955900  C -3.46836100 -1.21173600 1.86076500  C -1.80139900 -1.84195200 0.40479500  C -1.17265200 -0.84546500 1.23599800  C -2.29379000 -0.34762300 1.99127500  C -1.90761700 -1.47926900 -0.99691800  C -1.36292700 -0.18589700 -1.32518500  C -3.32626600 -1.58171000 -1.34597200  C -3.73978600 -0.28331600 -1.70720500  C -2.56234700 0.57553300 -1.56783600  C -4.09447100 -1.96968200 -0.20480200  C -5.10304900 -0.97123100 0.13203500  C -4.75605800 0.11076900 -0.78249700  C -4.59096900 -0.45675700 1.39695500  C -4.13850400 0.89462300 1.27554600  C -4.23907800 1.24739500 -0.08544600  C -2.87955800 1.54790700 -0.53712300  C -1.94526600 1.58355600 0.56045000  C -2.71898700 0.98809700 1.62007900  Zn -0.14077500 0.62355100 0.17000300  1 5 1.0 6 1.0 30 1.0 35 1.0  2 3 1.0 7 1.0 30 1.0 31 1.0  3 4 1.0 8 1.0 32 1.0  4 5 1.0 9 1.0 33 1.0  5 10 1.0 34 1.0  6  7  8  9  10  11 12 1.0 13 1.0 22 1.0 30 1.0  12 14 1.0 15 1.0 39 1.0  13 16 1.0 17 1.0 43 1.0  14 18 1.0 19 1.0 40 1.0  15  16 18 1.0 20 1.0 42 1.0  17  18 21 1.0 41 1.0  19  20  21  22 23 1.5 24 1.5  23 25 1.5 36 1.0  24 26 1.5 38 1.0  25 27 1.5 37 1.0  26 27 1.5 28 1.0  27 29 1.0  28  29  30 63 1.0  31  32  33  34  35  36  37  38  39  40  41  42  43  44 45 1.5 46 1.0 54 1.5  45 48 1.0 57 1.5  46 47 1.5 49 1.0  47 48 1.5 63 1.0  48 62 1.0  49 50 1.5 51 1.0  50 53 1.5 63 1.0  51 52 1.5 54 1.5  52 53 1.0 56 1.5  53 60 1.0  54 55 1.0  55 56 1.0 57 1.0  56 59 1.5  57 58 1.5  58 59 1.5 62 1.0  59 60 1.0  60 61 1.5  61 62 1.5 63 1.0  62  63 |
| AlC19 |
| %chk=AlC20.chk  %mem=40GB  %nprocshared=40  # opt uwb97xd/6-311+g(d,p) scrf=(cpcm,solvent=water) geom=connectivity  Title Card Required  0 2  C 0.88999100 -0.18364600 1.84885100  C 1.01887600 -1.38476200 1.12986600  C -0.54519800 0.16689300 1.85356700  C -1.30052300 -0.92237700 1.35996500  C -0.31688100 -1.78470200 0.72468100  C -0.67816400 1.43765400 1.11666400  C -1.60488900 1.52963000 0.00110500  C 0.66287500 1.80964800 0.70657400  C 0.66376600 1.81121700 -0.70237800  C -0.67653700 1.43996100 -1.11468800  C 1.61445600 0.79726000 1.11769900  C 2.30665800 0.21302800 0.00102300  C 1.61600400 0.79941100 -1.11442800  C 1.92462300 -1.20624500 -0.00065500  C 1.01965300 -1.38221700 -1.13254000  C 0.89170800 -0.18000500 -1.84896900  C -0.54332000 0.17119300 -1.85311700  C -1.29971900 -0.91947900 -1.36249200  C -0.31673500 -1.78256000 -0.72851900  Al -2.45845100 -0.19841800 -0.00101900  1 2 1.5 3 1.0 11 1.5  2 5 1.0 14 1.0  3 4 1.5 6 1.0  4 5 1.0 20 1.0  5 19 1.0  6 7 1.0 8 1.0  7 10 1.0 20 1.0  8 9 1.5 11 1.0  9 10 1.0 13 1.0  10 17 1.0  11 12 1.5  12 13 1.5 14 1.0  13 16 1.5  14 15 1.0  15 16 1.5 19 1.0  16 17 1.0  17 18 1.5  18 19 1.0 20 1.0  19  20 |
| AlC19@PCP |
| %chk=PCP-AlC19.chk  %mem=40GB  %nprocshared=40  # opt uwb97xd/6-311+g(d,p) scrf=(cpcm,solvent=water) geom=connectivity  Title Card Required  0 2  C 1.74999100 2.62461900 0.44471100  C 1.93863100 1.38331000 -1.65181400  C 1.56266900 2.74657400 -2.24262900  C 0.35721300 3.36342500 -1.54990100  C 0.68428800 3.59991900 -0.07035100  H 1.69564000 2.54094000 1.52859800  H 3.00090000 1.21888200 -1.80963800  H 2.41320500 3.42975500 -2.16351600  H 0.07391500 4.30104300 -2.03058800  H -0.21927100 3.54380600 0.53799200  C 2.66629500 0.25454300 0.51096900  C 4.04610100 0.92367600 0.67269200  C 2.14557900 -0.07803100 1.94223700  C 5.09150200 -0.02672700 1.28798500  H 4.42143500 1.34061200 -0.26379400  C 3.26306600 -0.26647800 2.98138200  H 1.47837700 0.71364400 2.29327800  C 4.47526100 -0.94578200 2.35414300  H 5.58201100 -0.62332300 0.51706000  H 2.86790000 -0.85454000 3.81246800  H 4.15903600 -1.89142900 1.90069000  C 2.75094300 -0.99704400 -0.38989000  C 3.75684400 -1.12869800 -1.35410700  C 1.80651900 -2.02214300 -0.31307300  C 3.80738900 -2.22311500 -2.20636500  C 1.84141200 -3.11433500 -1.17336600  C 2.84394100 -3.22210600 -2.12578500  H 1.08170400 -3.88263500 -1.08513200  H 2.87954200 -4.07479500 -2.79396300  N 1.64562500 1.24579700 -0.16236000  H 1.40421200 0.58298500 -2.16399900  H 1.37504400 2.60054600 -3.30799400  H -0.50863500 2.70099200 -1.65894500  H 1.09854200 4.60185600 0.06551300  H 2.73704200 3.01162200 0.19484100  H 4.52161600 -0.37088500 -1.46049200  H 4.60354200 -2.29028000 -2.93887000  H 1.01842800 -2.00091800 0.42543000  H 3.93094600 1.76852600 1.35168200  H 5.86683600 0.59764600 1.74009000  H 5.21871300 -1.19356700 3.11501400  H 3.56236700 0.70086600 3.39820800  H 1.54262900 -0.98202000 1.92263400  C -3.03904400 -2.13667600 0.88073200  C -3.31638800 -1.18314900 1.87584400  C -1.68263000 -1.83736800 0.36336700  C -1.06259700 -0.84791700 1.16090000  C -2.14180100 -0.33291600 1.98263900  C -1.83466300 -1.46406900 -1.04407500  C -1.26950900 -0.20510400 -1.49502400  C -3.25696100 -1.56250400 -1.34019500  C -3.67328100 -0.26240500 -1.68909200  C -2.48927200 0.57853500 -1.59123300  C -3.99459400 -1.93221000 -0.15133600  C -4.95812400 -0.92389600 0.22290100  C -4.65782900 0.13941600 -0.70758000  C -4.51879200 -0.43137700 1.53401700  C -3.98667700 0.90827600 1.31408600  C -4.13144300 1.27528500 -0.03554200  C -2.77204600 1.56930200 -0.55099500  C -1.83850500 1.57512000 0.51171800  C -2.56959800 1.00123200 1.62545000  Al -0.22218500 0.53807800 0.00841800  1 5 1.0 6 1.0 30 1.0 35 1.0  2 3 1.0 7 1.0 30 1.0 31 1.0  3 4 1.0 8 1.0 32 1.0  4 5 1.0 9 1.0 33 1.0  5 10 1.0 34 1.0  6  7  8  9  10  11 12 1.0 13 1.0 22 1.0  12 14 1.0 15 1.0 39 1.0  13 16 1.0 17 1.0 43 1.0  14 18 1.0 19 1.0 40 1.0  15  16 18 1.0 20 1.0 42 1.0  17  18 21 1.0 41 1.0  19  20  21  22 23 1.5 24 1.5  23 25 1.5 36 1.0  24 26 1.5 38 1.0  25 27 1.5 37 1.0  26 27 1.5 28 1.0  27 29 1.0  28  29  30 63 1.0  31  32  33  34  35  36  37  38  39  40  41  42  43  44 45 1.5 46 1.0 54 1.5  45 48 1.0 57 1.0  46 47 1.5 49 1.0  47 48 1.0 63 1.0  48 62 1.5  49 50 1.0 51 1.0  50 53 1.0 63 1.0  51 52 1.5 54 1.5  52 53 1.0 56 1.5  53 60 1.0  54 55 1.5  55 56 1.5 57 1.0  56 59 1.5  57 58 1.0  58 59 1.5 62 1.0  59 60 1.0  60 61 1.5  61 62 1.0 63 1.0  62  63 |
| C20 |
| %chk=C20.chk  %mem=40GB  %nprocshared=40  # opt rwb97xd/6-311+g(d,p) scrf=(cpcm,solvent=water) geom=connectivity  Title Card Required  0 1  C -0.74842900 -1.94932400 0.03040600  C -1.15838100 -1.16724300 1.13443700  C 0.78215100 -1.93688600 0.02597200  C 1.18511200 -1.14691800 1.12748000  C 0.01159500 -0.68834300 1.81732200  C 1.17996800 -1.18481700 -1.10072700  C 1.96145300 0.00624200 -0.70011900  C 0.00110700 -0.74649400 -1.79633200  C -0.01133100 0.68838800 -1.81723200  C 1.15849700 1.16731600 -1.13466600  C -1.16568900 -1.20438100 -1.09446300  C -1.96722400 -0.02733500 -0.69030000  C -1.18512900 1.14690800 -1.12756200  C -1.96157000 -0.00620200 0.70024100  C -1.18019000 1.18468100 1.10074600  C -0.78228700 1.93676700 -0.02593800  C 0.74814800 1.94904400 -0.03027800  C 1.16575200 1.20480800 1.09457200  C -0.00106500 0.74637700 1.79617000  C 1.96751300 0.02741000 0.69027300  1 2 1.5 3 1.0 11 1.5  2 5 1.5 14 1.0  3 4 1.5 6 1.5  4 5 1.5 20 1.0  5 19 1.5  6 7 1.0 8 1.5  7 10 1.0 20 1.5  8 9 1.5 11 1.5  9 10 1.5 13 1.5  10 17 1.5  11 12 1.0  12 13 1.0 14 1.5  13 16 1.5  14 15 1.0  15 16 1.5 19 1.5  16 17 1.0  17 18 1.5  18 19 1.5 20 1.0  19  20 |
| C20@PCP |
| %chk=PCP-C20.chk  %mem=40GB  %nprocshared=40  # opt rwb97xd/6-311+g(d,p) scrf=(cpcm,solvent=water) geom=connectivity  Title Card Required  0 1  C 0.99570200 2.44078300 0.63488500  C 1.18322100 1.43591000 -1.64493800  C 2.17675600 2.51848500 -2.06101500  C 2.13412600 3.75542600 -1.14818500  C 0.92254200 3.68973100 -0.22618000  H 0.16702500 2.39624500 1.33603700  H 1.36307800 0.49274200 -2.14628900  H 3.19157100 2.13833000 -2.16223300  H 3.03773300 3.79805000 -0.53279000  H -0.01507500 3.73047400 -0.78728200  C 2.11454800 0.13477600 0.44650400  C 3.51328800 0.76063400 0.25756300  C 1.86129500 0.00921200 1.97943800  C 4.62536200 -0.15561700 0.80100100  H 3.72514700 0.96981700 -0.78470700  C 3.14930800 -0.02838700 2.81885100  H 1.25269700 0.83504000 2.33750700  C 4.24241400 -0.83264900 2.12743800  H 4.88500700 -0.90740500 0.05270100  H 2.90683500 -0.45282300 3.79564200  H 3.87747000 -1.84766300 1.94258500  C 2.09211000 -1.21921100 -0.29542300  C 2.67801200 -1.34137300 -1.56311300  C 1.59087900 -2.38716200 0.27967500  C 2.69493400 -2.54760100 -2.24841700  C 1.61533400 -3.60265700 -0.39391200  C 2.15296800 -3.68857600 -1.66934800  H 1.20618300 -4.48292000 0.08827600  H 2.16799700 -4.63429400 -2.19876500  N 1.00291100 1.12881500 -0.16065000  H 0.19986100 1.77056800 -1.96513800  H 1.86192500 2.78920900 -3.07209500  H 2.11518700 4.66933900 -1.74333800  H 0.91344900 4.53716300 0.46310800  H 1.91049900 2.48306300 1.21607600  H 3.15515400 -0.49460400 -2.03819000  H 3.14833600 -2.59417000 -3.23170500  H 1.15882000 -2.38373000 1.26861700  H 3.56752600 1.71627000 0.77842300  H 5.51137200 0.46911100 0.93963400  H 5.11893300 -0.92598100 2.77234700  H 3.50763100 0.99004000 3.00425600  H 1.27622700 -0.87631700 2.19874100  C -3.06329700 -1.04699700 -1.59416800  C -2.69276000 -2.05714700 -0.65815600  C -1.92184000 -0.16489100 -1.79947000  C -0.83452600 -0.59552500 -1.06299200  C -1.38017400 -1.72044100 -0.26646900  C -2.33594700 1.21671600 -1.42545700  C -1.55856400 1.59522600 -0.36459900  C -3.72010700 1.09876500 -0.93168500  C -3.74996300 1.46299100 0.43256100  C -2.42552700 1.81015700 0.82751300  C -4.12705100 -0.27234500 -1.02176800  C -4.54182400 -0.79012700 0.27019900  C -4.26233000 0.29264700 1.21960900  C -3.57414100 -1.81843400 0.54854000  C -2.79359200 -1.49737100 1.67276900  C -3.20127100 -0.11963900 2.02749100  C -2.06978000 0.76012300 1.72407500  C -0.97665000 -0.00184000 1.23170800  C -1.44893900 -1.32514300 1.10061800  C -0.43634600 0.55033900 -0.09295500  1 5 1.0 6 1.0 30 1.0 35 1.0  2 3 1.0 7 1.0 30 1.0 31 1.0  3 4 1.0 8 1.0 32 1.0  4 5 1.0 9 1.0 33 1.0  5 10 1.0 34 1.0  6  7  8  9  10  11 12 1.0 13 1.0 22 1.0  12 14 1.0 15 1.0 39 1.0  13 16 1.0 17 1.0 43 1.0  14 18 1.0 19 1.0 40 1.0  15  16 18 1.0 20 1.0 42 1.0  17  18 21 1.0 41 1.0  19  20  21  22 23 1.5 24 1.5  23 25 1.5 36 1.0  24 26 1.5 38 1.0  25 27 1.5 37 1.0  26 27 1.5 28 1.0  27 29 1.0  28  29  30 63 1.0  31  32  33  34  35  36  37  38  39  40  41  42  43  44 45 1.5 46 1.0 54 1.5  45 48 1.5 57 1.0  46 47 2.0 49 1.0  47 48 1.0 63 1.0  48 62 1.5  49 50 2.0 51 1.0  50 53 1.0 63 1.0  51 52 1.5 54 1.5  52 53 1.5 56 1.0  53 60 1.5  54 55 1.0  55 56 1.0 57 1.5  56 59 1.5  57 58 1.5  58 59 1.0 62 1.0  59 60 1.0  60 61 1.5  61 62 1.5 63 1.0  62  63 |
